# Photoreceptor degeneration in *ABCA4*-associated retinopathy and its genetic correlates

**DOI:** 10.1172/jci.insight.155373

**Published:** 2022-01-25

**Authors:** Maximilian Pfau, Catherine A. Cukras, Laryssa A. Huryn, Wadih M. Zein, Ehsan Ullah, Marisa P. Boyle, Amy Turriff, Michelle A. Chen, Aarti S. Hinduja, Hermann E.A. Siebel, Robert B. Hufnagel, Brett G. Jeffrey, Brian P. Brooks

**Affiliations:** 1 National Eye Institute, National Institutes of Health, Bethesda, Maryland, USA.; 2 Department of Ophthalmology, University of Bonn, Bonn, Germany.

**Keywords:** Ophthalmology, Genetic diseases, Retinopathy

## Abstract

**BACKGROUND:**

Outcome measures sensitive to disease progression are needed for ATP-binding cassette, sub-family A, member 4–associated (*ABCA4*-associated) retinopathy. We aimed to quantify ellipsoid zone (EZ) loss and photoreceptor degeneration beyond *EZ-loss* in *ABCA4*-associated retinopathy and investigate associations between photoreceptor degeneration, genotype, and age.

**METHODS:**

We analyzed 132 eyes from 66 patients (of 67 enrolled) with molecularly confirmed *ABCA4*-associated retinopathy from a prospective natural history study with a median [IQR] follow-up of 4.2 years [3.1, 5.1]. Longitudinal spectral-domain optical coherence tomography volume scans (37 B-scans, 30° × 15°) were segmented using a deep learning (DL) approach. For genotype-phenotype analysis, a model of *ABCA4* variants was applied with the age of criterion *EZ-loss* (6.25 mm^2^) as the dependent variable.

**RESULTS:**

Patients exhibited an average (square-root-transformed) *EZ-loss* progression rate of [95% CI] 0.09 mm/y [0.06, 0.11]. Outer nuclear layer (ONL) thinning extended beyond the area of *EZ-loss*. The average distance from the *EZ-loss* boundary to normalization of ONL thickness (to ±2 *z* score units) was 3.20° [2.53, 3.87]. Inner segment (IS) and outer segment (OS) thinning was less pronounced, with an average distance from the *EZ-loss* boundary to layer thickness normalization of 1.20° [0.91, 1.48] for the IS and 0.60° [0.49, 0.72] for the OS. An additive model of allele severity explained 52.7% of variability in the age of criterion *EZ-loss*.

**CONCLUSION:**

Patients with *ABCA4*-associated retinopathy exhibited significant alterations of photoreceptors outside of *EZ-loss*. DL-based analysis of photoreceptor laminae may help monitor disease progression and estimate the severity of *ABCA4* variants.

**TRIAL REGISTRATION:**

ClinicalTrials.gov identifier: NCT01736293.

**FUNDING:**

National Eye Institute Intramural Research Program and German Research Foundation grant PF950/1-1.

## Graphical abstract

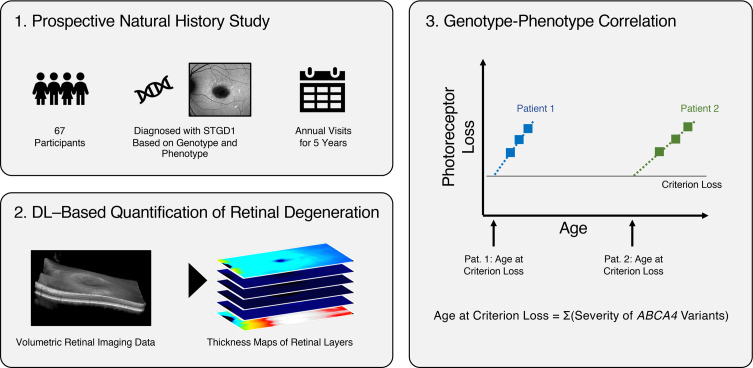

## Introduction

Stargardt disease (Stargardt disease-1, STGD1; MIM #248200) is the most common cause of inherited macular dystrophy, with a prevalence between 1 in 8000 to 10,000 (1). This autosomal-recessive disease is caused by mutations in the *ABCA4* gene (ATP-binding cassette, sub-family A, member 4; OMIM #601691) coding for transmembrane transporter protein (2).

STGD1 is characterized by macular yellow, pisciform flecks that typically spare the peri-papillary retina and centrifugally progressing atrophy of the outer retina. Both the age of onset and the spatial pattern of STGD1 disease are highly variable (3). Patients may present clinically with a wide range of phenotypes, including early-onset cone-rod dystrophy (3), juvenile-onset retinal dystrophy affecting predominantly foveal function, or late-onset STGD1 with foveal sparing (4–6). This heterogeneity complicates the choice of outcome measures applicable across this broad phenotypic spectrum.

The end stages of degeneration are marked by retinal pigment epithelium (RPE) atrophy, and progression of these areas is being measured by short-wavelength hypoautofluorescence (hypoFAF) (7, 8). Changes in hypoFAF area are currently being used as the primary outcome measure for a clinical trial involving Stargardt disease (i.e., ClinicalTrials.gov NCT03772665). Functionally, the boundary of deep scotomata often exceeds the margins of RPE atrophy in STGD1 (9). In early stages of disease, markers of disease progression have also been proposed, including the leading disease front as observed in the distribution of flecks (10), and lipofuscin accumulation measured by (quantitative) autofluorescence imaging (11–13). Functionally, these structural changes precede retinal sensitivity loss. Quantification of photoreceptor loss in terms of ellipsoid zone (EZ) loss progression in spectral-domain optical coherence tomography (SD-OCT) has been proposed (14) and currently serves as the outcome measure for 2 clinical trials of Stargardt disease (i.e., ClinicalTrials.gov NCT04545736, NCT03364153). To date, long-term data on the progression pattern of *EZ-loss* in STGD1 in a large cohort are lacking.

At the level of an individual A-scan, quantification of *EZ-loss* represents a binary metric (i.e., each pixel on an en face map EZ is either absent or present). As such, information pertaining to more subtle degeneration of photoreceptors beyond the boundaries of *EZ-loss* is not captured.

Moreover, *EZ-loss* is not ideal for evaluating disease severity in the *Abca4*^−/−^ mouse model due to the limited axial resolution of OCT imaging in mice. Thus, direct comparison of experimental studies in animal models and human data is challenging (15). As a step toward a unified measure of disease progression in *ABCA4*-related retinopathy that could apply to human and animal models, thinning the outer nuclear layer may be a potential candidate. A large study by Whitmore and colleagues examined thinning of retinal layers (16). However, their analysis was limited to the fixed Early Treatment Diabetic Retinopathy Study grid (*ETDRS-grid*) across patients, which resulted in the systematic exclusion of patients with progressed disease (16). Accordingly, the development of a framework to analyze the progression of photoreceptor degeneration beyond the boundaries of *EZ-loss* across patients with different degrees of disease severity is desired.

Quantitative analysis of photoreceptor laminae thinning in *ABCA4*-related retinopathy may provide an opportunity to classify allele severity in an interval-scaled manner analogous to a previous perimetry-based study (17). Such a quantitative metric of variant severity would be invaluable for preclinical studies examining specific variants (4, 17–19).

The purpose of this study was to quantify and compare photoreceptor degeneration and its change over time in terms of (i) *EZ-loss* progression, (ii) *ETDRS-grid*-based thinning of photoreceptor laminae, and (iii) photoreceptor laminae thinning outside of *EZ-loss* using a potentially novel, individualized, contour line–based approach. In addition, (iv) this study provides a framework to impute the age of criterion *EZ-loss* (on the level of each eye) to quantify the severity of individual *ABCA4* variants in an interval-scaled manner.

## Results

### Cohort.

From a total of 67 study participants, 132 eyes from 66 patients were included in this analysis (40 female [60.6%], 26 male [39.4%], Table 1). SD-OCT volume scans could not be obtained from 1 study participant with poor fixation nasal to the optic disc; this participant was excluded from the present analysis (Supplemental Figure 1; supplemental material available online with this article; https://doi.org/10.1172/jci.insight.155373DS1).

A median [IQR] follow-up of 4.2 years [3.1, 5.1] was available for the participants in terms of annual study visits in addition to an initial 6-month retest visit. Further SD-OCT data acquired (with the same settings) before or after the study were also available (cf. Methods for details). Since linear mixed models can handle unbalanced repeated measures data, these additional SD-OCT data were also included in the analyses. With these additional visits, the overall median follow-up time was 5.0 years [3.4, 6.1]. Throughout this article, the first visit refers to the first visit with imaging data available, while baseline refers to the baseline visit of the prospective natural history study (Supplemental Figure 2).

At baseline, the median [IQR] acuity for the better eyes was 0.8 logMAR [0.36, 0.96] (approx. 20/125 [20/50, 20/200] Snellen equivalent) and 0.86 logMAR [0.52, 1.00] (approx. 20/160 [20/63, 20/200] Snellen equivalent) for the worse eyes. Best corrected visual acuity worsened slightly over time with a rate of (mixed model estimate [95% CI]) 0.01 logMAR/y [0.01–0.02] (Supplemental Figure 3).

### Progression of EZ-loss.

**Figure 1:**
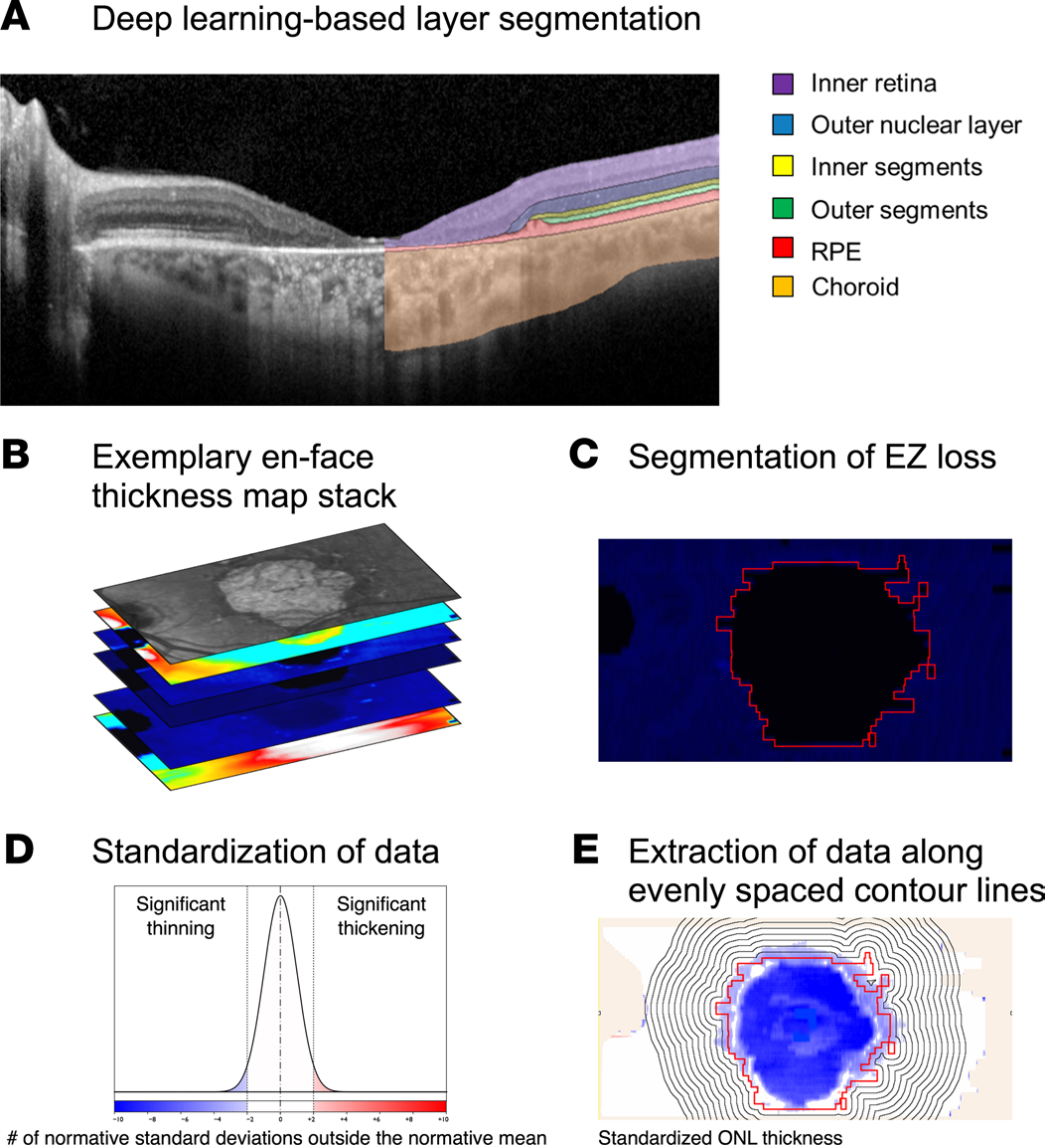
Feature extraction. (**A**) Six retinal layers were segmented using a convolutional neural network. (**B**) Subsequently, en face projections were generated for each layer. (**C**) The area of ellipsoid zone (EZ) loss (shown in red) could be identified on the photoreceptor outer segment (OS) thickness map. (**D** and **E**) To account for age and retinal topography, the retinal thickness data for each A-scan (i.e., pixel in the en face map) were normalized in a pointwise manner using normal data as *z* score. (**E**) Retinal layer thicknesses in relation to the EZ boundary were extracted along evenly spaced contour lines (0.43° between the contour lines). RPE, retinal pigment epithelium.

A deep learning–based (DL-based) pipeline allowed for automated generation of photoreceptor thickness maps and segmentation of *EZ-loss* based on the complete absence of photoreceptor outer segments (Figure 1). Validation of the layer segmentation in B-scans of eyes, which were not applied for model training, showed good agreement with manual annotations (Supplemental Figure 4). Likewise, validation against manually segmented *EZ-loss* indicated overall good agreement (intraclass correlation coefficient of 0.91, 95% limits of agreement of –1.3 mm^2^ and 1.4 mm^2^), with little to no bias (mean difference estimate [95% CI] of 0.03 mm^2^ [–0.16, 0.22], cf. Supplemental Figure 5).

**Figure 2:**
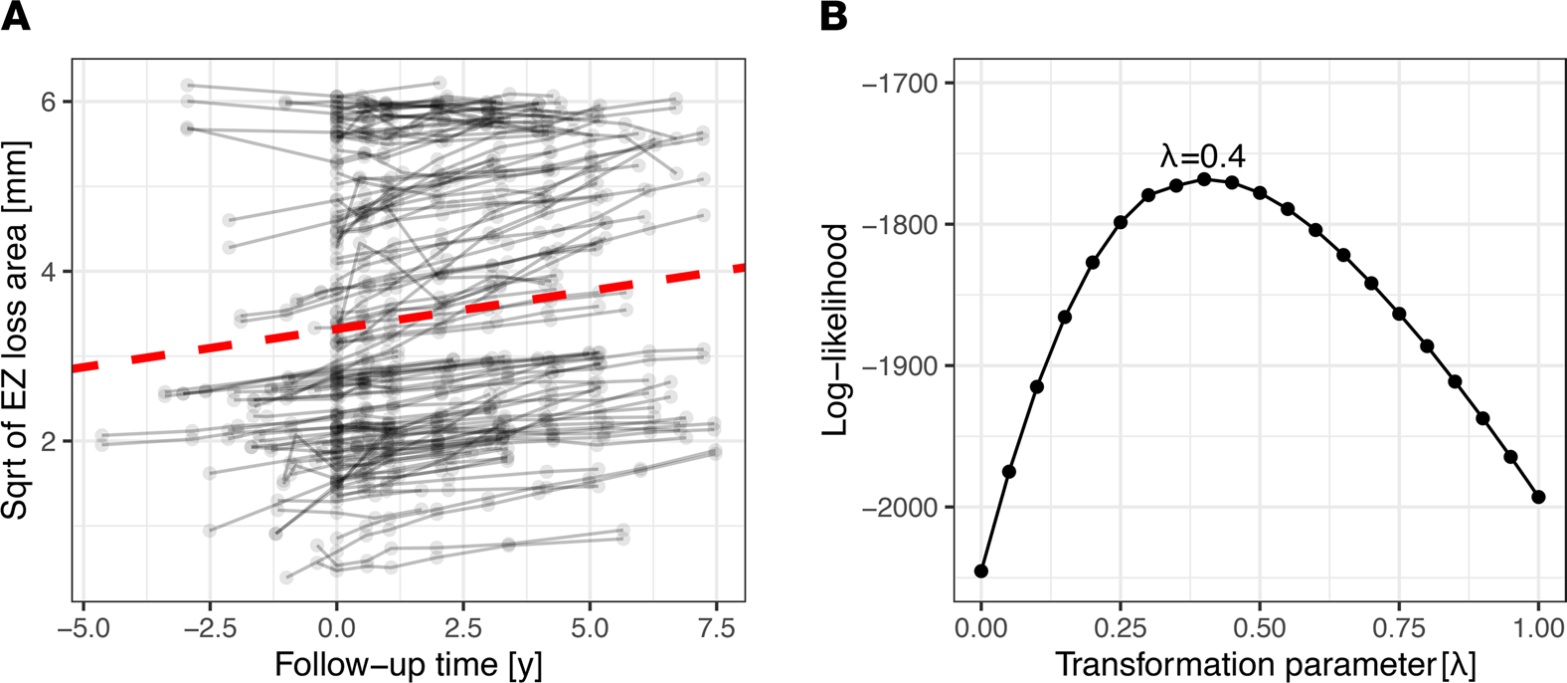
Progression of *EZ-loss*. (**A**) The first panel shows the square-root-transformed progression of *EZ-loss* over time (with a rolling median filter, span: ±1 year). The red dashed line shows the mixed model estimate for *EZ-loss* progression. (**B**) The second panel shows the log-likelihood (*y* axis) for mixed models of *EZ-loss* (dependent variable) as a function of time (independent variable). Models were fit with a range of Box-Cox transformations (λ 0 to 1) of the dependent variable *EZ-loss*. Based on the log-likelihood (45), a Box-Cox transformation parameter λ of 0.4 was optimal, which approximates square-root transformation (i.e., Box-Cox λ of 0.5). Supplemental Figure 9 shows the *EZ-loss* progression for exemplary eyes. These plots include data acquired prior to the baseline visit of the natural history study up to the last visit of each patient (*N* of patients = 66).

Figure 2A shows the progression of the *EZ-loss* area over time. The rate of progression was approximately linear after the square-root transformation of the *EZ-loss* area. Using a “nonparametric profile maximum likelihood” approach, a Box-Cox transformation with λ = 0.4 was optimal to achieve normality of the response distribution in a random-effects model (Figure 2B). A similar transformation of the data can be achieved using a square-root transformation of the area of *EZ-loss* (i.e., Box-Cox transformation with a λ = 0.5). A square-root transformation corresponds to the linear progression of *EZ-loss* along the radius of the lesion and is thus an intuitive transformation (20). As a result, square-root transformation of the area of *EZ-loss* was applied for subsequent analyses. Supplemental Figure 9 shows the *EZ-loss* progression for exemplary eyes.

The average (square-root transformed) *EZ-loss* area at baseline was (mixed-model estimate [95% CI]) 3.32 mm [2.93, 3.71] with an annual progression rate of 0.09 mm/y [0.06, 0.11] (*P* < 0.001, Figure 2A). The spread of the square-root-transformed *EZ-loss* progression rate was wide (median [IQR] of 0.07 mm/y [0.03, 0.12], Supplemental Figure 6), which translates to a spread for the absolute *EZ-loss* progression rate of (median [IQR]) 0.39 mm^2^/y [0.16, 0.7].

### Retinal layer thickness outside of EZ-loss.

**Figure 3:**
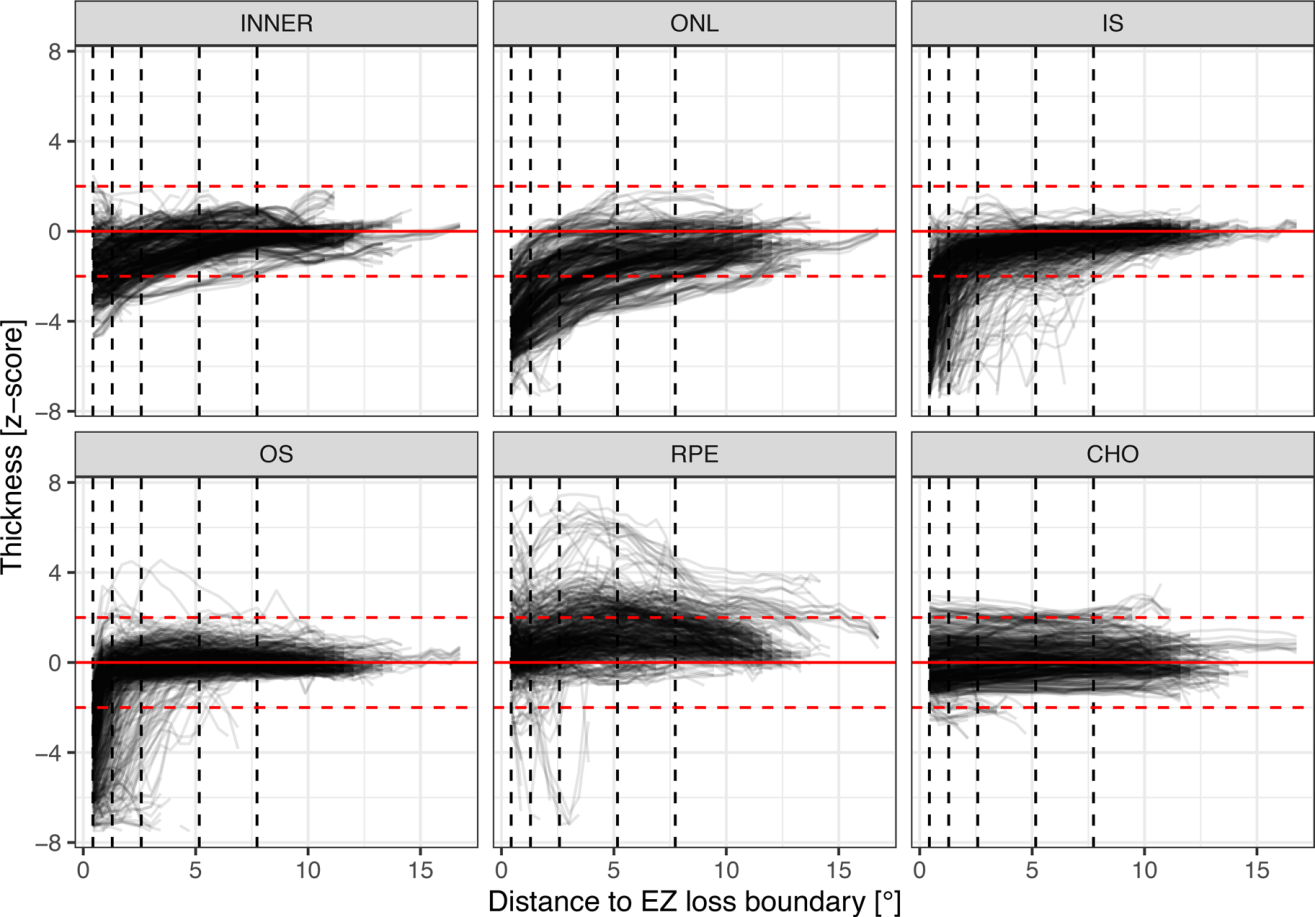
Retinal layer abnormalities outside the area of ellipsoid zone (EZ) loss. The line plots show the normalized retinal layer thicknesses (*y* axis) outside the area of *EZ-loss* in eyes with STGD1 as a function of the distance to the *EZ-loss* boundary (*x* axis). The horizontal, red, dashed lines denote ±2 *z* score units (i.e., the normative range). The vertical, black, dashed lines indicate the distances 0.43°, 1.29°, 2.58°, 5.16°, and 7.73° (multiples of a Goldmann III stimulus diameter) to the *EZ-loss* boundary, which correspond to the average distance to normalization of thickness for the inner retina (INNER), outer nuclear layer (ONL), and photoreceptor inner segments (IS) and outer segments (OS). For these distances, changes over time in layer thicknesses are shown in Figure 4. Of note, INNER, ONL, IS, and OS are all severely thinned even outside the area of *EZ-loss*. The retinal pigment epithelium (RPE) shows thickening outside of *EZ-loss*. For the choroid (CHO), no marked changes in terms of thickness are evident. These plots are based on the data from the baseline of the natural history up to the last visit of each patient (*N* of patients = 66).

Figure 3 shows normalized retinal layer thickness as a function of the distance from the boundary of *EZ-loss* for each patient. The distance from *EZ-loss* to the contour line, where retinal layer thickness normalized on average (i.e., thickness within ±2 *z* score units), varied markedly between the layers (Figure 3). The average distance for normalization was 1.07° [0.85, 1.30] for the inner retina, 3.20° [2.53, 3.87] for the ONL, 1.20° [0.91, 1.48] for the IS, 0.60° [0.49, 0.72] for the OS, and 0.63° [0.42, 0.83] for the RPE.

At the first visit, the inner retina, ONL, IS, and OS were significantly thinned at the contour line directly outside of *EZ-loss* (0.43° contour line). Specifically, the inner retina was thinned by (mixed model estimate [95% CI]) –1.54 *z* score units [–1.81, –1.26], the ONL by –3.60 *z* score units [–3.93, –3.27], IS by –3.34 *z* score units [–4.17, –2.51], and OS by –2.11 *z* score units [–2.53, –1.69] (all *P* < 0.001). In contrast, the RPE was slightly thickened directly outside of *EZ-loss* along the 0.43° contour line with +0.61 *z* score units [0.29, 0.94] (*P* < 0.001). The average choroidal thickness was within normal limits at 0.00 *z* score units [–0.23, 0.23] (*P* = 0.992) (Figure 3). Only 4 eyes of 2 patients with severe neuroretinal atrophy exhibited statistically significant choroidal thinning (i.e., below –2 *z* score units, Figure 3). In terms of thickness deviation, these values translate to –23.69 μm [–28.14, –19.24] for the inner retina, –31.51 μm [–34.63, –28.40] for the ONL, –6.50 μm [–8.22, –4.78] for the IS, –6.46 μm [–7.75, –5.18] for the OS, +1.97 μm [0.90, 3.05] for the RPE, and 1.21 μm [–19.53, 21.96] for the choroid.

### Progression of photoreceptor degeneration outside of EZ-loss.

**Figure 4:**
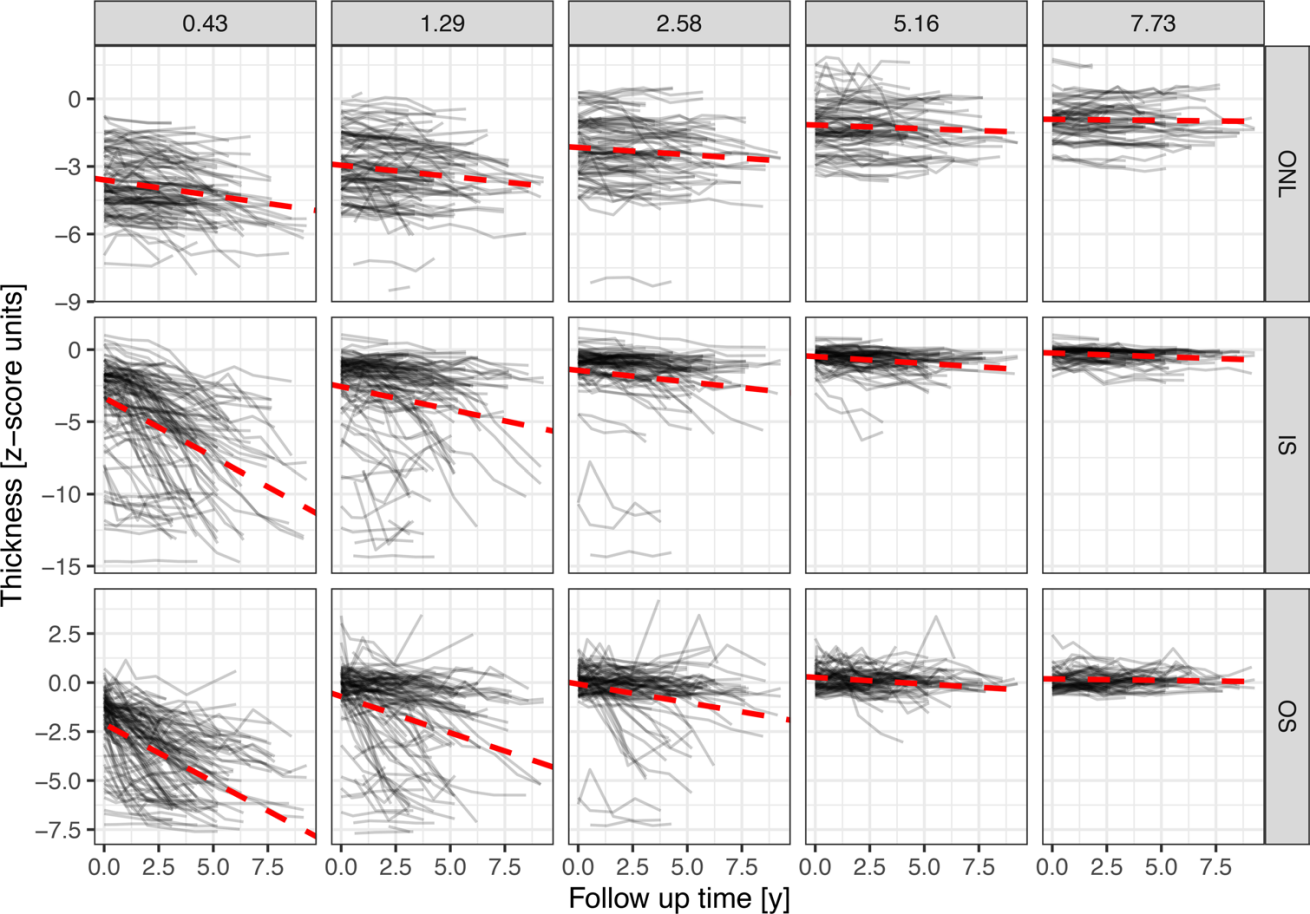
Rate of change in layer thickness per year. The parallel line plots show the change in normalized layer thicknesses (*y* axis) over time (*x* axis) as a function of the distance to the *EZ-loss* boundary at baseline (panels). Each line denotes data from an individual eye. The red dashed lines are derived from mixed model estimates for the change over time. Of note, there is no evidence of “retina-wide” photoreceptor loss in this cohort. These plots are based on the data from baseline of the natural history up to the last visit of each patient (*N* of patients = 66).

Figure 4 shows the rate of change in layer thickness over time as a function of the distance to the *EZ-loss* boundary at the first visit. All 3 photoreceptor laminae exhibited significant thinning over time in the immediate junctional zone. The thinning rate was greatest at the 0.43° contour line and less at more distant contour lines. Directly outside of the *EZ-loss* boundary, the rate of change (mixed model estimate [95% CI]) was –0.14 *z* score units/y [–0.18, –0.10] for the ONL thickness, –0.82 *z* score units/y [–0.99, –0.65] for the IS thickness, and –0.59 *z* score units/y [–0.69, –0.49] for the OS thickness. These values correspond in terms of micrometers to a rate of change at the 0.43° contour line of –1.23 μm/y [–1.58, –0.88] for the ONL, –1.41 μm/y [–1.70, –1.13] for the IS, and –1.67 μm/y [–1.95, –1.40] for the OS.

At more distant contour lines, the change over time was overall lower (Figure 4). At the 7.73° contour line, ONL did not thin significantly over time (–0.01 *z* score units per year [–0.03, 0.01], *P* = 0.431), IS exhibited slight but significant change (–0.05 *z* score units /y [–0.07, –0.03], *P* < 0.001), and OS exhibited no change (–0.01 *z*score units/y [–0.05, 0.02], *P* = 0.383). Supplemental Tables 1 and 2 describe the change in layer thickness over time across eccentricities in terms of the *z* score (i.e., adjusted for spatial differences in the variability of normal layer thicknesses) and in micrometers (i.e., “unadjusted” for spatial differences in the variability of normal layer thicknesses), respectively.

### Progression of retinal degeneration within ETDRS subfields.

*ETDRS-grid*-based analysis revealed approximately linear thinning over time for all segmented retinal layers except for the inner retina in the central ETDRS subfield (Supplemental Figures 7 and 8). For the central ETDRS subfield, ONL thinned by an average of (mixed model estimate [95% CI]) –1.22 μm/y [–1.85, –0.59], IS by –0.4 μm/y [–0.65, –0.16], OS by –0.15 μm/y [–0.26, –0.05], RPE by –0.51 μm/y [–0.85, –0.17], and choroid by –6.71 μm/y [–8.49, –4.93]. The estimates for retinal layer thinning in the inner ETDRS subfields were overall similar (Supplemental Table 3). Of note, *ETDRS-grid*-based analysis resulted in marked floor effects. Especially within the central ETDRS subfield, many patients exhibited severe degeneration across layers at baseline, with the result that no or only minimal progression occurred over time (Supplemental Figures 7 and 8). Overall, observed progression rates of photoreceptor degeneration (ONL, IS, OS thinning) were markedly larger when quantified in an individualized manner (along contour lines in proximity to the *EZ-loss* boundary, Supplemental Table 2) compared with the conventional (spatially fixed) *ETDRS-grid*-based analysis (Supplemental Table 3).

### Genetic determinants of photoreceptor degeneration.

Given the linearity of the square-root-transformed EZ-loss progression rate, the age at which each eye reached (or will have reached) an *EZ-loss* area of 6.25 mm^2^ (square-root-transformed = 2.5 mm) was estimated (Supplemental Figure 9). Below, this estimate is referred to as the age of criterion *EZ-loss*. This (arbitrary) criterion was selected since square-root-transformed *EZ-loss* progression rates were linear in this value range (cf. Figure 2). This time-invariant estimate of disease severity reflects both the age of onset and subsequent rate of progression. Notably, estimates of the age of criterion *EZ-loss* showed a strong intrapatient correlation (*R^2^* of 90.7%, Supplemental Figure 10 and Supplemental Table 4).

With the assumption that each *ABCA4* variant has an independent, additive contribution to the age of criterion *EZ-loss*, (17) it was possible to fit a linear (mixed-effects) model to the data (Supplemental Methods Section 1). Table 2 shows the ages of criterion *EZ-loss* (y) derived from the model for 31 variants from 43 patients (Supplemental Methods Section 1 and Supplemental Figure 1). For a patient with 2 null variants, the model predicted that the age of criterion *EZ-loss* would be 13.76 years (i.e., 6.88 years + 6.88 years). By comparison for a patient with p.Gly1961Glu and null variants, the model predicts an age of onset of 41.51 years (i.e., 34.63 years + 6.88 years). The model explained (marginal *R^2^*) 52.7% of the variability in age of criterion *EZ-loss* (Table 2).

To validate the additive model, we used leave-one-out cross-validation in a subset of 23 patients who had an overlap of both variants with other patients (Supplemental Methods Section 2 and Supplemental Figure 1). In this subset, the model could be fitted iteratively on *n* – 1 patients and then be evaluated iteratively with the withheld patient (leave-one-out cross-validation). Despite the rather small sample size available for model fitting (23 – 1 patients), the models explained (leave-one-out cross-validated *R^2^*) 24.1% of the variability in the age of criterion *EZ-loss* (Supplemental Figure 11).

**Figure 5:**
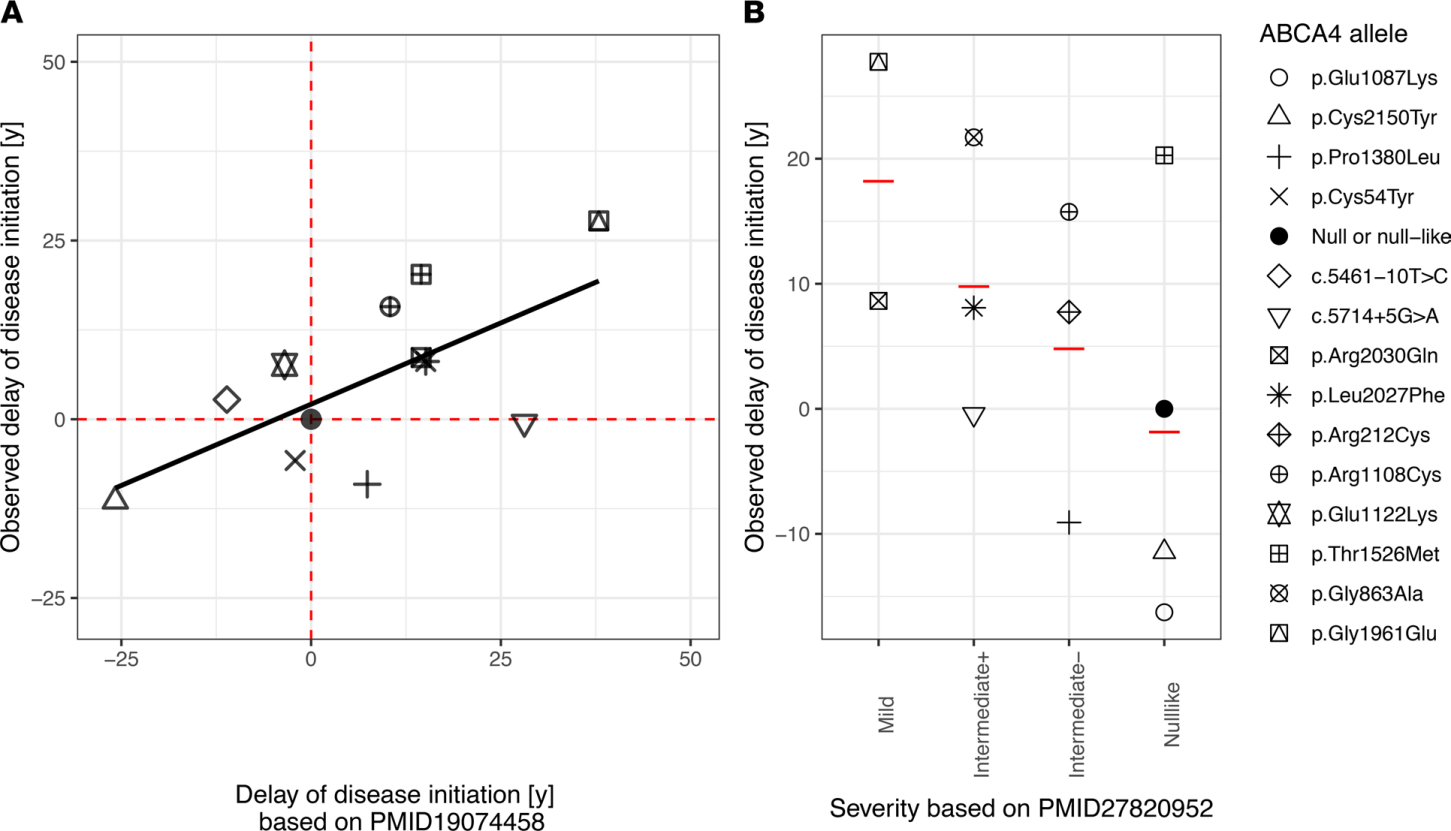
Comparison of the allele severity estimates with prior publications. (**A**) The first panel shows the comparison to overlapping data on 12 variants from Cideciyan et al. 2009 (17). The *x* axis shows the delay of disease initiation relative to a null mutation as estimated by Cideciyan et al. 2009. The *y* axis shows the estimates for the delay of disease initiation relative to a null mutation in the present study (age of criterion *EZ-loss* minus 6.88 years [estimate for age of criterion *EZ-loss* for a null mutation]). Interestingly, these prior data explained (*R*^2^) 43.5% of the variability in the delay of disease initiation observed in our data. (**B**) The second panel shows the comparison to overlapping data on 12 variants from Fakin et al. 2016 (4). The *x* axis shows the ordinal-scaled classification from Fakin et al. 2016. The *y* axis shows the estimates for the delay of disease initiation relative to a null mutation in the present study. The red horizontal lines indicate the median in the observed delay of disease initiation for each category.

For external validation, we compared the results from our additive model to the previously published interval-scaled (visual field–based) classification of variant variability by Cideciyan and coworkers, who used retinal sensitivity data as a readout (17). Twelve variants common to both works were compared. These prior estimates of variant severity showed a moderate correlation with our estimates of variant severity with an *R^2^* of 43.5% (Figure 5A). Similarly, 12 variants were overlapping with the ordinal (electrophysiology-based) classification by Fakin and coworkers (4). Our interval-scaled estimates of disease severity mostly agreed with this prior classification (Figure 5B).

## Discussion

This study provides a detailed analysis of the progression of photoreceptor loss over time using SD-OCT in *ABCA4*-associated retinopathy. Integral to this analysis, we developed and validated a potentially novel method that quantified changes in the thickness of the retinal layers with time along contours equidistant to the *EZ-loss* boundary. Analysis along a contour line closest to the *EZ-loss* boundary showed clear evidence of disease progression that was not evident from conventional (“spatially fixed”) *ETDRS-grid*-based analysis. In addition, an *EZ-loss*-based approach to define a time-invariant measure of disease severity was proposed. This time-invariant estimate of the age of criterion *EZ-loss*, which reflects age of onset and subsequent progression rate, allowed generating a (hypothetical) interval-scaled classification for the severity of 31 *ABCA4* variants.

Sensitive structural outcome measures for STGD1 are an essential prerequisite for therapeutic trials. Currently, ongoing therapeutic trials apply the area of definitely decreased autofluorescence (DDAF, e.g., SeaSTAR [NCT03772665]), area of *EZ-loss* (STAR [NCT03364153], National Eye Institute [NEI] metformin trial [NCT04545736]), or quantitative autofluorescence (STARTT [EudraCT No. 2018-001496-20]) as outcome measures. While DDAF in STGD1 is well established in terms of reproducibility and progression rates (8, 21), the boundaries of absolute scotomata may exceed the boundaries of DDAF in STGD1 (in contrast to age-related macular degeneration) (9, 22). Thus, the application of DDAF as a surrogate of disease severity in STGD1 may represent the trailing edge of the disease. In comparison, photoreceptor integrity and *EZ-loss* were previously shown to correlate closely with retinal light sensitivity (23) and electrophysiological characteristics (24). Moreover, *EZ-loss* is well established as an outcome measure across retinal diseases (25, 26). We propose progression of photoreceptor loss in the retina immediately surrounding the area of *EZ-loss* represents a measure of the leading front of the disease and, as such, may prove a valuable outcome measure for clinical trials of *ABCA4*-related retinopathy.

Overall, our prospective cohort’s *EZ-loss* progression rates were readily quantifiable and compatible with prior estimates from smaller retrospective cohorts (14, 27). Similar to these previous series, we note that different patients sometimes have disparate rates of change. However, analogous to earlier reports for DDAF (28, 29), square-root transformation of the *EZ-loss* area resulted in a linear trend over time. This indicates that the progression of the *EZ-loss* area is linear along the radius, which may have important implications for predicting the rate of change for each individual. Concerning change over time, the here-observed annual progression rate of 0.09 mm/y for the *EZ-loss* area closely matched previous estimates of DDAF progression (7, 28). Thus, even though the *EZ-loss* area exceeds the area of RPE atrophy (10), both disease fronts appear to progress in parallel. Given the linearity of square-root-transformed progression rates, it was possible to compute the age of criterion *EZ-loss* as a time-invariant measure of disease severity for each patient. This time-invariant estimate of age of disease initiation (ADI) allowed us to generate a hypothetical interval-scaled classification for the severity of 31 *ABCA4* variants.

As a prerequisite to the genotype-phenotype correlation, we could compute the age of criterion *EZ-loss* with high reliability, as underscored by the strong correlation of the age of criterion *EZ-loss* predicted separately for the left and right eyes of patients (Supplemental Figure 10). Similarly, Lambertus and coworkers (30), and Tanna and coworkers (14), previously evidenced a strong intereye correlation in lesion size and progression of atrophy in STGD1 as measured by fundus autofluorescence and EZ band loss, respectively. Our interval-scaled classification for severity exhibited an overall moderate agreement with Cideciyan and coworkers’ (perimetry-based, interval-scaled) (17), as well as Fakin and coworkers’, prior classifications (electrophysiology-based, ordinal-scaled) (4). For example, these and other prior publications have classified the p.Gly1961Glu variant as a mild variant, typically associated with a bull’s eye maculopathy phenotype and paucity of flecks (4, 17, 31). The common *ABCA4* variant c.5461-10T>C was estimated to have a similar effect on the age of criterion *EZ-loss* as null mutations in our data, which is compatible with previously published data (17, 32, 33).

Meanwhile, at the severe end of the spectrum, p.Cys2150Tyr was predicted to be associated with an earlier age of criterion *EZ-loss* than a null variant. This estimate is again in line with the data from Cideciyan and colleagues, who also considered this variant to be more severe than a null variant (17). Fakin and colleagues classified this variant as “null-like.” The effect of these types of variants, which are associated with a disease onset earlier than null variants, cannot be explained by mere loss of gene function. It has been previously suggested that the severity of recessive diseases, including STGD1, can be modified by toxic gain of function (17, 34). The implications of these variants for therapeutic trials are unclear to date. However, it is conceivable that patients with putative toxic gain-of-function variants may benefit from gene replacement therapy to a lesser extent (or perhaps not at all). These putative toxic gain-of-function variants also highlight a major shortcoming of the less granular, ordinal-scaled variant classifications, (4, 18) which do not provide a distinct class for variants more severe than “null-like.”

For some variants, our data are in disagreement with prior observations. For example, *ABCA4* c.5714+5G>A was not associated with a delayed age of criterion *EZ-loss* compared to null variants in our data. However, this variant was previously shown to be associated with a marked delay of disease initiation by Cideciyan et al. and classified as “intermediate +” by Fakin et al. (4, 17). Considering this variant as milder than “null-like” is further supported by an in vitro splice assay showing that 39.8% of the *ABCA4* c.5714+5G>A transcripts are correctly spliced (32). Another variant with discrepant results was *ABCA4* p.Thr1526Met. This variant was associated with a delayed disease initiation in the study of Cideciyan et al. and our study but classified as “null-like” by Fakin and colleagues (4, 17).

Disagreement in the severity classification of variants can originate from 3 primary sources. First, the selected severity metric (age of criterion *EZ-loss*, perimetry-based sensitivity loss, vs. electrophysiological characteristics) may affect the results. Second, the additive model is (most likely) a simplification, and interaction effects between variants might result in a more or less severe phenotype than predicted by the simple sum of the allele severities (35). Last, unobserved genetic variation, which encompasses variants within or outside the *ABCA4* gene, could alter the disease severity (36). The suspected female predilection of STGD1 can be considered as an example of genetic variation outside of the *ABCA4* gene, which is associated with disease severity (37).

A notable finding of the present study was that we could not observe photoreceptor degeneration over time at a distance of 7.73° to the *EZ-loss* boundary of the first visit. We expected to observe ONL thinning distant to the boundary of *EZ-loss* in a subset of patients given retinal sensitivity and electroretinogram (ERG) findings from previous studies of *ABCA4*-related retinopathy. Approximately half of the *ABCA4* patients in an earlier study using wide-field perimetry exhibited abnormal extramacular cone and rod sensitivity that progressed over time (1.1 log/decade and 0.45 log/decade) (17). Likewise, in a separate study, *ABCA4* patients with abnormal cone and rod ERG amplitudes (Lois Group III; ref. 38) at baseline exhibited an amplitude attenuation of –3.6%/y on dark-adapted 11.0 A-wave and 3.1%/y on light-adapted 30 Hz flicker (38). Our results seemingly conflict with these earlier studies, assuming that photoreceptor loss is the source of retinal sensitivity loss in *ABCA4*-associated retinopathy (11) and that ONL thickness closely correlates with photoreceptor density in animal models (39). Potentially, the inclusion criteria of this study (especially ability to perform SD-OCT imaging with averaged scan) excluded patients who would have shown “peripheral” ONL degeneration at the boundary of the scans. The electrophysiological characteristics of our cohort support this notion. Only 10 (15%) of the right eyes of the 67 included patients had at baseline a scotopic ERG B-wave amplitude outside the normal limits.

Using a DL-based image segmentation pipeline, this study allowed us to quantify photoreceptor degeneration in *ABCA4*-associated retinopathy in a large, genetically well-characterized cohort. However, the en face imaging frame was limited (30° × 15°), which led to the inability to evaluate *EZ-loss* progression in a small subset of patients (ceiling effect). Based on previous data, the en face B-scan density (distance of 120 μm) used here was sufficient for the accurate quantification of photoreceptor thinning (40). However, floor effects were evident regarding the progression of IS and OS thinning. For these thin laminae, a higher B-scan density and ultra-high-resolution OCT will be required to assess change over time more accurately (40, 41). The genotype-phenotype analysis was based on a single imputed metric (age of criterion *EZ-loss*). As noted above, the genotype-phenotype analysis was based on an additive model. Thus, it could not reflect potential interaction effects. In addition, unobserved genetic variation within and outside of the *ABCA4* gene may further influence disease severity.

In summary, we have demonstrated the application of a DL-based pipeline to characterize photoreceptor degeneration over time in *ABCA4*-associated retinopathy. This approach allowed us to evaluate in a fully automated manner the progression of conventional biomarkers (e.g., ETDRS-based analysis of photoreceptor laminae thinning), as well as contour line–based analysis of photoreceptor degeneration over time. In addition, we demonstrated that the age of *EZ-loss* is dependent on the genotype and provided estimates for 31 variants, including 16 variants, which we believe have not been previously quantitatively analyzed regarding clinical severity (Supplemental Table 4).

## Methods

### Patients.

Patients included in this analysis participated in a noninterventional, prospective, longitudinal natural history for STGD1 conducted at the NEI (ClinicalTrials.gov NCT01736293).

Sixty-seven patients were recruited between October 2012 and September 2018. Study visits included baseline, 6 months, 1 year, and then yearly visits for 5 years. To be included in this study, STGD1 had to be confirmed based on the clinical phenotype, and presence of at least 1 pathogenic *ABCA4* mutation, and patients had to be 12 years or older. Exclusion criteria were evidence of a systemic condition or ocular disease unrelated to *ABCA4* mutations that would complicate the analysis of psychophysical, electrophysiological, or imaging data (e.g., diabetic eye disease). Lesion size or visual acuity did not constitute inclusion or exclusion criteria in this study.

Previously acquired normal data over a wide age range (imaging of patients’ companions and or patients with a healthy fellow eye) were included to account for normal aging and retinal topography (42).

### Imaging protocol.

Patients underwent 30° × 30° fundus autofluorescence imaging (λ excitation, 488 nm; λ emission, 500–700 nm), 30° × 30° infrared reflectance (λ 815 nm) imaging, and 30° × 15° SD-OCT imaging (37 B-scans, automatic real-time tracking of 25) using a Spectralis HRA+OCT (Heidelberg Engineering).

### Analysis set.

One study patient had to be excluded from the presented analysis due to the lack of SD-OCT volume scans (Supplemental Figure 1). Prebaseline imaging data were available for a subset of patients that were acquired with the same settings as in the main study. These prebaseline imaging data were also included in the analysis to obtain more accurate estimates of change over time. Throughout this article, the first visit refers to the first visit with imaging data available, while baseline refers to the baseline visit of the study (Supplemental Figure 2).

### DL-based image segmentation.

Retinal layer segmentations were obtained using a previously validated convolutional neural network (42). For the SD-OCT B-scan multilayer segmentation (Figure 1), the same layer definitions were applied for the inner retina, ONL, photoreceptor IS, photoreceptor OS, and RPE as in a previous study (42). Importantly, Henle’s fiber layer and hyporeflective wedge-shaped bands at the boundary of atrophy were consistently counted toward the ONL to facilitate reproducible annotation (42, 43). The RPE band definition included the RPE and flecks, again in consideration of the interrater variability. Following B-scan-wise segmentation, en face thickness maps were generated for all retinal layers. Based on the loss of OS, the area of *EZ-loss* was segmented (Figure 1).

Finally, the thickness data were standardized in an A-scan-wise manner (conversion to *z* scores) to account for age and location-specific variation of normal thickness.

Regions with vignetting artifacts were excluded from the analysis. Likewise, the peri-papillary retina (circular area with a radius of 5° centered to the optic disc) was excluded from the analysis given the previously described unique characteristics of the peri-papillary retina in STGD1 (44).

### Feature extraction.

For each visit, we extracted the area of *EZ-loss* (mm^2^), as well as retinal layer thicknesses (both absolute values [μm] and standardized [*z* scores]) along evenly spaced contour lines surrounding the *EZ-loss* boundary (spacing of 0.43° between the contour lines [i.e., multiples of a Goldmann III stimulus diameter], Figure 1).

### Manual segmentation of EZ band.

The length of central discontinuity of the EZ band was measured manually for each SD-OCT B-scan collected. All measurements were made manually, with each B-scan examined at 800% zoom using the 1:1 μm setting. The area of EZ band loss, *AreaEZ_loss_* (mm^2^), was calculated from the Riemann sum:

where *X_k_* (μm) = the length of central EZ band discontinuity of the *k*^th^ B-scan and *ΔX* (μm) = the distance between B-scans. The start of the intact EZ band was sometimes obvious; more frequently, there was an ambiguous region between the obvious absence or presence of the EZ band. To account for this uncertainty, we applied the following rules to determine the edge of the intact EZ band for each B-scan: starting at the peripheral edge of the scan and moving toward the fovea, the start of the discontinuity was defined as the first evidence of loss of the EZ band. Small breaks in the EZ band (<250 μm) were not counted as loss if there was a section of continuous EZ band measuring at least 200 μm, closer to the fovea. This last condition was designed to disregard disruptions due to flecks, which we observed to be generally <225 μm in size and transitory with time, i.e., presence of a fleck at 1 time point did not mean the absence of EZ band at a subsequent time point.

### Statistics.

Statistical analyses were performed in the software environment *R*. Normal distributed data were summarized by their mean and standard deviation; non-normal data were summarized by their median and IQR. A *P* value less than 0.05 was considered statistically significant.

The optimal λ for Box-Cox transformation to model the area of *EZ-loss* over time with mixed-effects models was identified using the *R* package *boxcoxmix* (45). The optimal λ for the Box-Cox transformation (in terms of the maximum likelihood estimator) was close to the λ value that produces a square-root transformation (see Results, Figure 1). Thus, we used a square-root transformation of the area of *EZ-loss* for all subsequent analyses. For all subsequent analyses of longitudinal data, linear mixed models (random intercept and slope models) were applied with eye nested in patients as random effects terms using the *R* package *lme4* (46). *P* values were obtained using Satterthwaite’s approximation.

For genotype-phenotype correlation, the age at which *EZ-loss* reached (or was expected to have reached) a predefined criterion *EZ-loss* area (6.25 mm^2^) was imputed for each eye. This estimated age of criterion *EZ-loss* provided an age-invariant variable that could be compared to the genotype. Specifically, a linear model was fit to the (square-root) transformed *EZ-loss* progression data from each eye and applied to infer the age at which the eye was expected to have had a square-root-transformed *EZ-loss* area of 2.5 mm (6.25 mm^2^, Supplemental Figure 9). This size criterion was chosen since we could document the linearity of square-root-transformed *EZ-loss* progression in this value range. In patients with *EZ-loss* area progression reaching the limits of the image frame during the study (>16 mm^2^), the *EZ-loss* progression rate was determined based on the first 2 visits. For the analysis, an additive model (mixed model) of *ABCA4* variants was fitted to the data with the age of criterion *EZ-loss* as a dependent variable analogous to the ADI analysis previously proposed by Cideciyan and coworkers (cf. Supplemental Methods Section 1 for details) (17). Only patients (*n* = 43) with exactly 2 *ABCA4* variants and a measurable *EZ-loss* area in at least 2 visits could be included in this analysis (Supplemental Figure 1).

A subset of patients (*n* = 23), with 2 *ABCA4* variants that both occurred with other patients in this study, was used to assess the accuracy of the modeling approach through patient-wise leave-one-out cross-validation (cf. Supplemental Methods Section 2 for details).

### Study approval.

This study adhered to the tenets of the Declaration of Helsinki and was approved by the institutional review board of the National Institutes of Health. Written informed consent was obtained from all participants prior to inclusion in the study. No compensation/incentive was offered to the participants.

## Author contributions

CAC, WMZ, AT, BGJ, and BPB contributed to research design. CAC, LAH, WMZ, and BPB contributed to data acquisition. MP, BGJ, RBH, EU, MPB, HEAS, ASH, and MAC contributed to analysis and interpretation of data. MP, CAC, LAH, WMZ, RBH, EU, BGJ, and BPB contributed to drafting of the manuscript.

## Supplementary Material

Supplemental data

Trial reporting checklists

ICMJE disclosure forms

## Figures and Tables

**Table 1:**
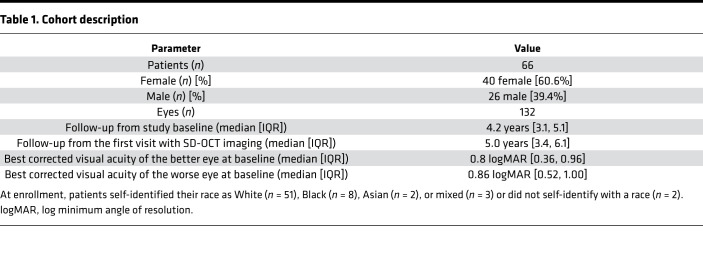
Cohort description

**Table 2:**
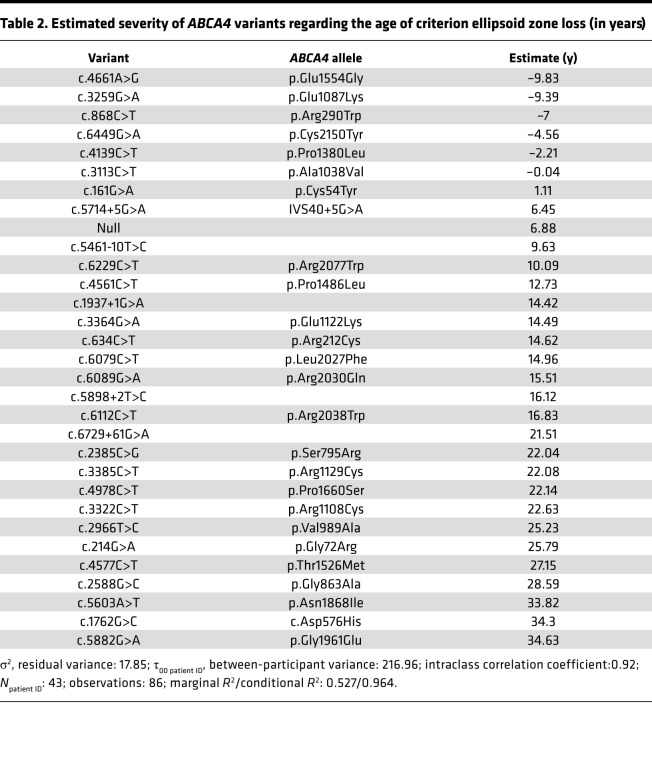
Estimated severity of *ABCA4* variants regarding the age of criterion ellipsoid zone loss (in years)

## Version 1 01/25/2022

Electronic publication
