# Supplementary material for: Photoreceptor degeneration in ABCA4-associated retinopathy and its genetic correlates
Source: JCI Insight. 2022 Jan 25;7(2):e155373. doi: 10.1172/jci.insight.155373 (PMC8855828; doi:10.1172/jci.insight.155373)
Supplement: Supplemental data [file jciinsight-7-155373-s142.pdf]

Supplemental Data

Supplemental data to: **Photoreceptor Degeneration in *ABCA4*-associated Retinopathy and its Genetic Correlates**

Maximilian Pfau,1,2 Catherine A. Cukras,1 Laryssa A. Huryn,1 Wadih M. Zein,1 Ehsan Ullah,1 Marisa P. Boyle,1 Amy Turriff,1,* Michelle A. Chen,1 Aarti S. Hinduja,1 Hermann Siebel,1 Robert B. Hufnagel,1 Brett G. Jeffrey,1,† Brian P. Brooks1,†

Supplementary Figure S1. Selection of Patients for Genotype-Phenotype Analysis

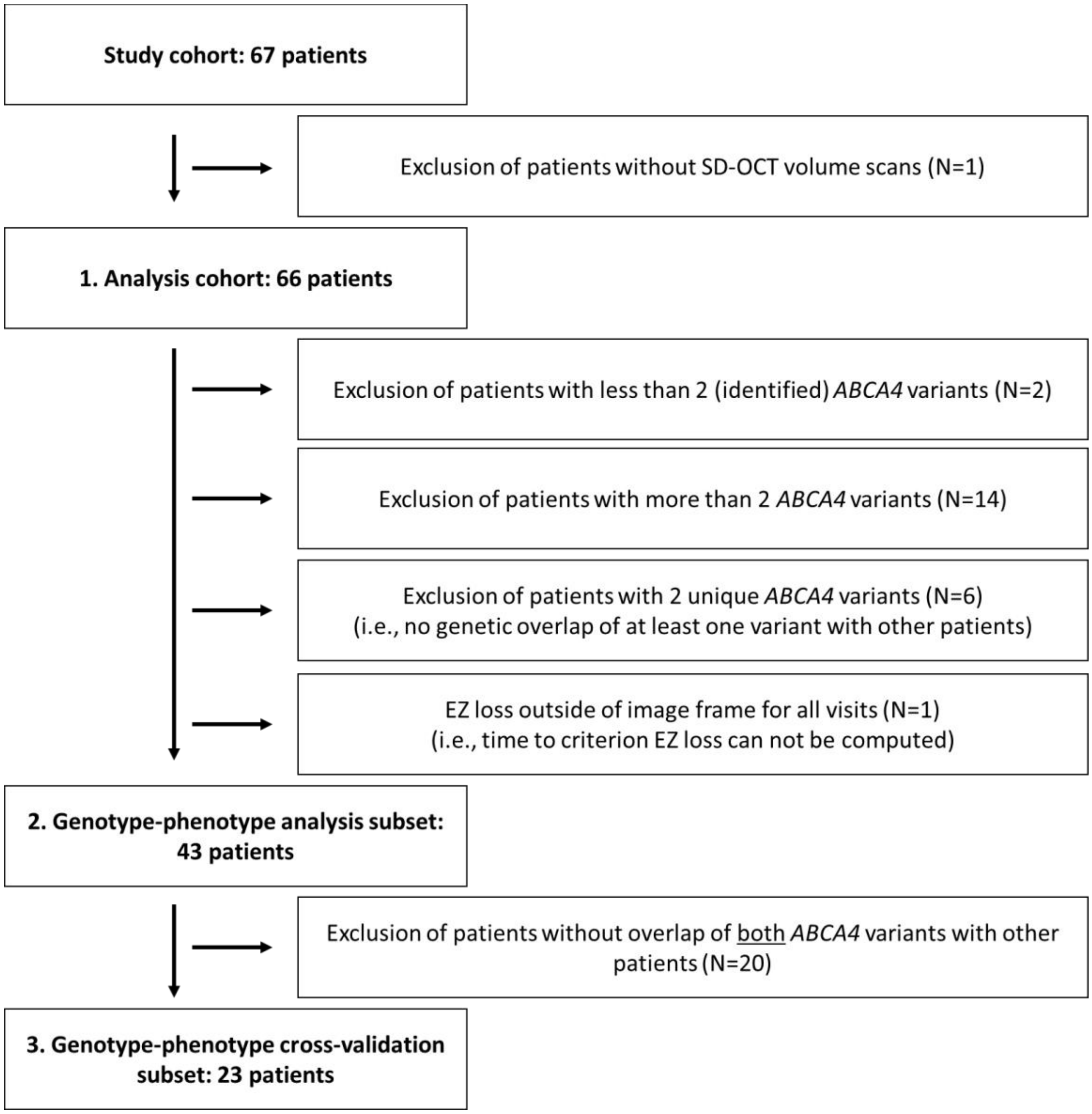

Supplementary Figure S2. Overview of available imaging data

The dot plot denotes for each eye (y-axis) the available imaging data over time (x-axis). Since linear mixed models, which can handle unbalanced data, were applied for all analyses, imaging data prior to and following the prospective natural history study visits were included. All optical coherence tomography scans were acquired using identical settings in terms of area and number of B-scans.

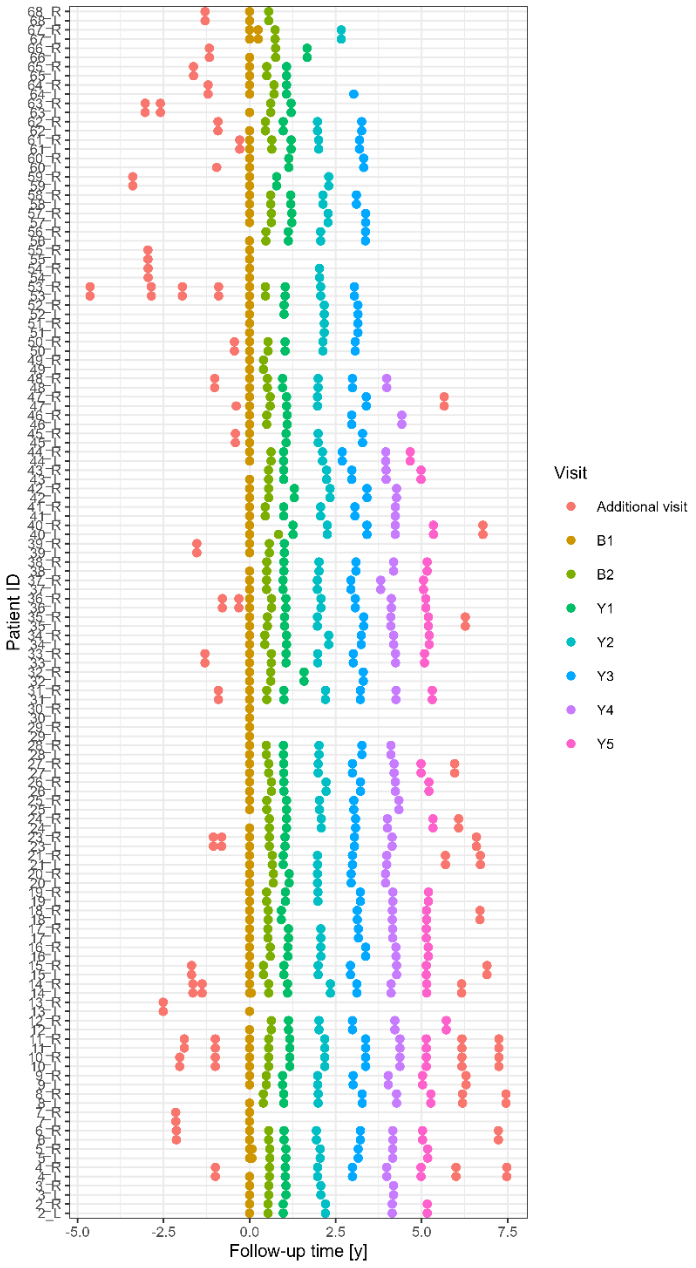

Supplementary Figure S3. Best-corrected visual acuity

(**A**) The panel shows the change in best-corrected visual acuity over time, which was statistically significant, but small in magnitude (mixed model estimate [95% CI] of 0.01 LogMAR/yr [0.01 – 0.02]).

(**B**) The panel shows the association of best-corrected visual acuity as a function of the log10 transformed outer nuclear layer (ONL) thickness in the central subfield (CSF) of the ETDRS grid. The red dashed trend line was generated using locally estimated scatterplot smoothing (LOESS).

These plots are based on the data of from baseline of the natural history study to the year 5 follow-up visit (N of patients = 66).

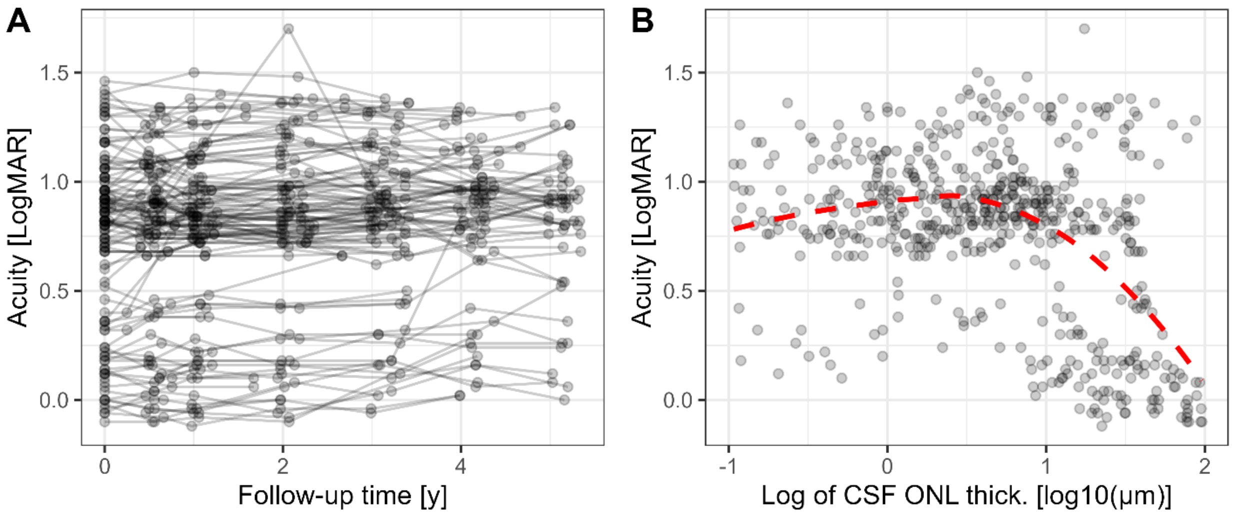

Supplementary Figure S4. Validation of the retinal layer segementation

An independent test set (i.e., data, which was not used for the training and/or hyper-parameter optimization of the segementation algorithm) of 3 B-scans from 15 eyes was manually segmented to assess the performance of the segmentation algorithm. The central and two extrafoveal B-scans were selected for each patient.

The Dice similarity coefficient (F1 Score) as a measure of overlap was used to assess quantiatfely the segmentation perfromance. The dot plot shows the results for the individual B-scans and the red dots and errorbars denote the mean Dice coeffcient and 95% confidence interval. The dependencies (B-scan nested in patient) were considered in the computation of the 95% confidence intervals.

The Dice coefficent was (mean estimate ± SE) 0.97 ±0.01 for the background, 0.96 ±0.01 for the inner retina, 0.79±0.05 for the ONL, 0.76 ±0.05 for the IS, 0.76 ±0.04 for the OS, 0.82 ±0.06 for the RPE, and 0.89 ±0.01 for the CHO.

Abbreviations: inner retina (INNER), outer nuclear layer (ONL), photoreceptor inner segments (IS), photoreceptor outer segments (OS), retinal pigment epithelium (RPE), choroid (CHO)

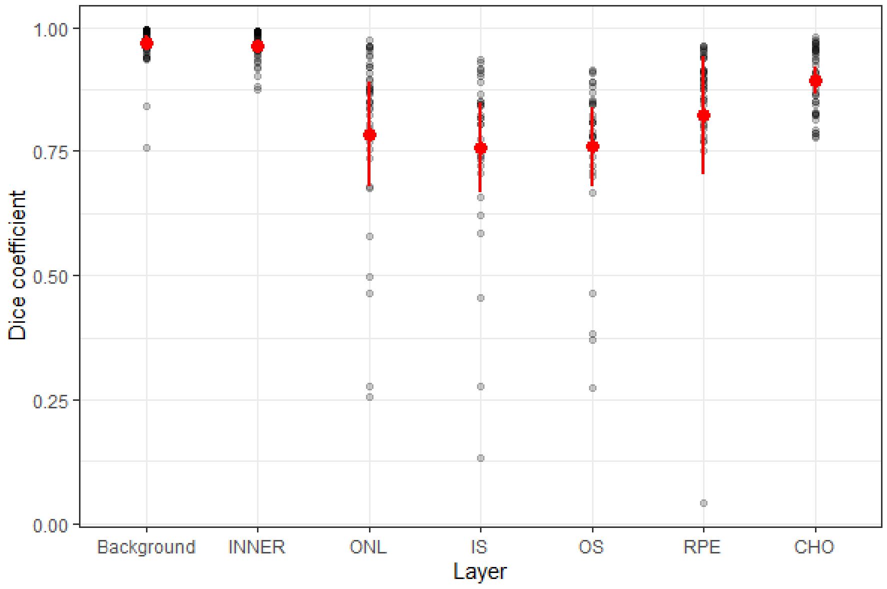

Supplementary Figure S5. Validation of ellipsoid zone (EZ) loss segmentation

Manually segmented data for 360 visits from 54 eyes of 27 patients were available for validation of the segmentation model. These visits were not used for training of the segmentation model. Linear mixed models were applied to compute the Bland-Altman indices, while accounting for the data structure (repeated measurements in eyes nested in patients)

(**A** and **B**) The upper panels show the comparison between the deep learning (DL)-based results for the central lesion versus the manual segmentation. (**A**) The Bland Altman plot shows that the fully-automated method exhibited no bias (mean difference estimate [95% CI] of 0.03 mm^2 [-0.16, 0.22], solid red line) with good agreement among the measurements (95% limits of agreement [LoAs] of -1.3 mm^2 and 1.4 mm^2, indicated by the dashed lines). (**B**) The resulting slope estimates (for the square-root transformed *EZ-loss* progression) were similar among both methods with no bias (mean difference [95% CI] of 0.01 mm/yr [0.00, 0.02], 95% LoAs of -0.06 mm/yr and 0.08 mm/yr).

(**C**) shows the comparison of the DL-based central *EZ-loss* (i.e., main lesion) versus the total *EZ-loss* (includes main lesion and *EZ-loss* overlying flecks). The central *EZ-loss* tended to underestimate the total *EZ-loss* area slightly (mean difference of 0.20 mm^2 [0.09, 0.30]).
(**D**) These differences in absolute *EZ-loss* area had little to no effect on the slope estimates.

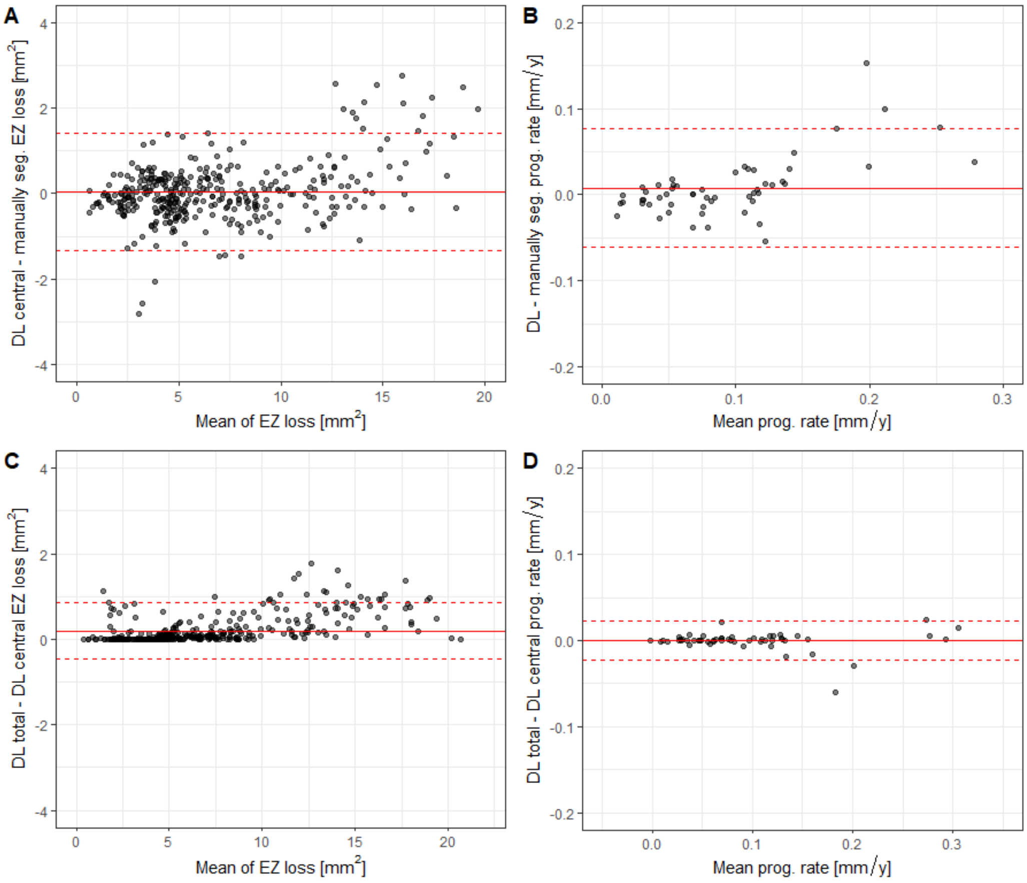

Supplementary Figure S6. Ranked plot of the (ellipsoid zone) *EZ-loss* progression rate

The dot plot denotes for each eye (y-axis) the square-root transformed *EZ-loss* progression rate. Due to the limitation of the image frame and minor segmentation deviations, negative *EZ-loss* progression could occur, which reflects the genuine uncertainty of the method. No post-hoc transformations were applied to enforce monotonic trends (i.e., no application of a running max filter). Vertical dashed red line shows average annual progression rate of 0.09 mm/yr

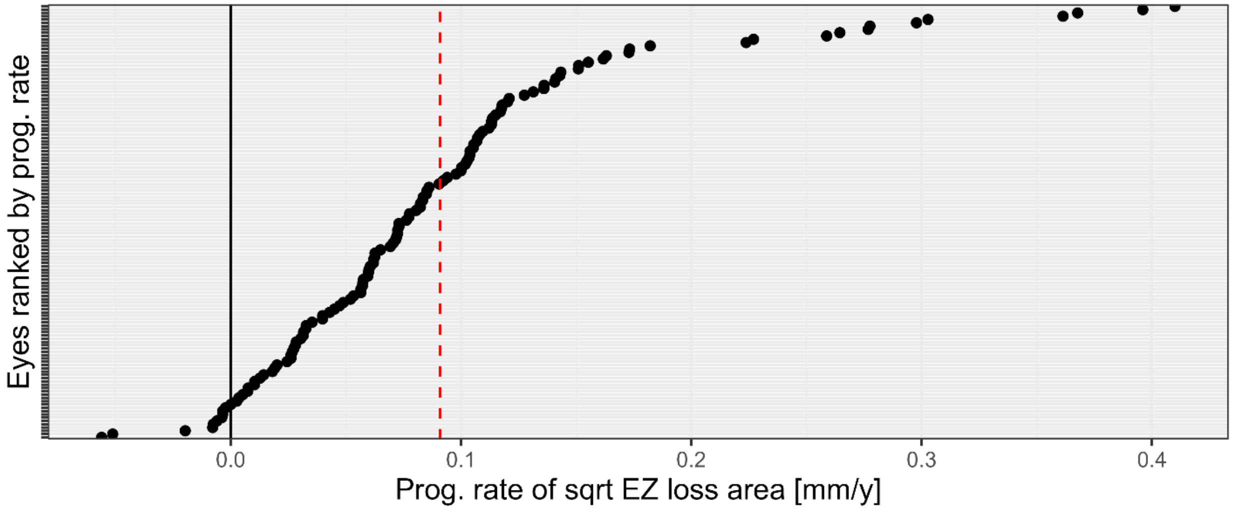

Supplementary Figure S7. Progression of photoreceptor degeneration within ETDRS subfields

The upper sketch shows the optic nerve head and the *ETDRS-grid* centered to the fovea with a central subfield (CSF) diameter of 1 mm and four inner subfields extending from 0.5 mm to 1.5 mm. The line plots show the change in layer thicknesses (μm, y-axis) over time (x-axis) as a function of the retinal location (ETDRS subfields, panels).

These plots include data acquired prior to the baseline visit of the natural history study up to the last visit of each patient (N of patients = 66).

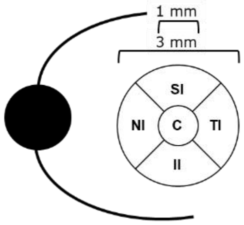

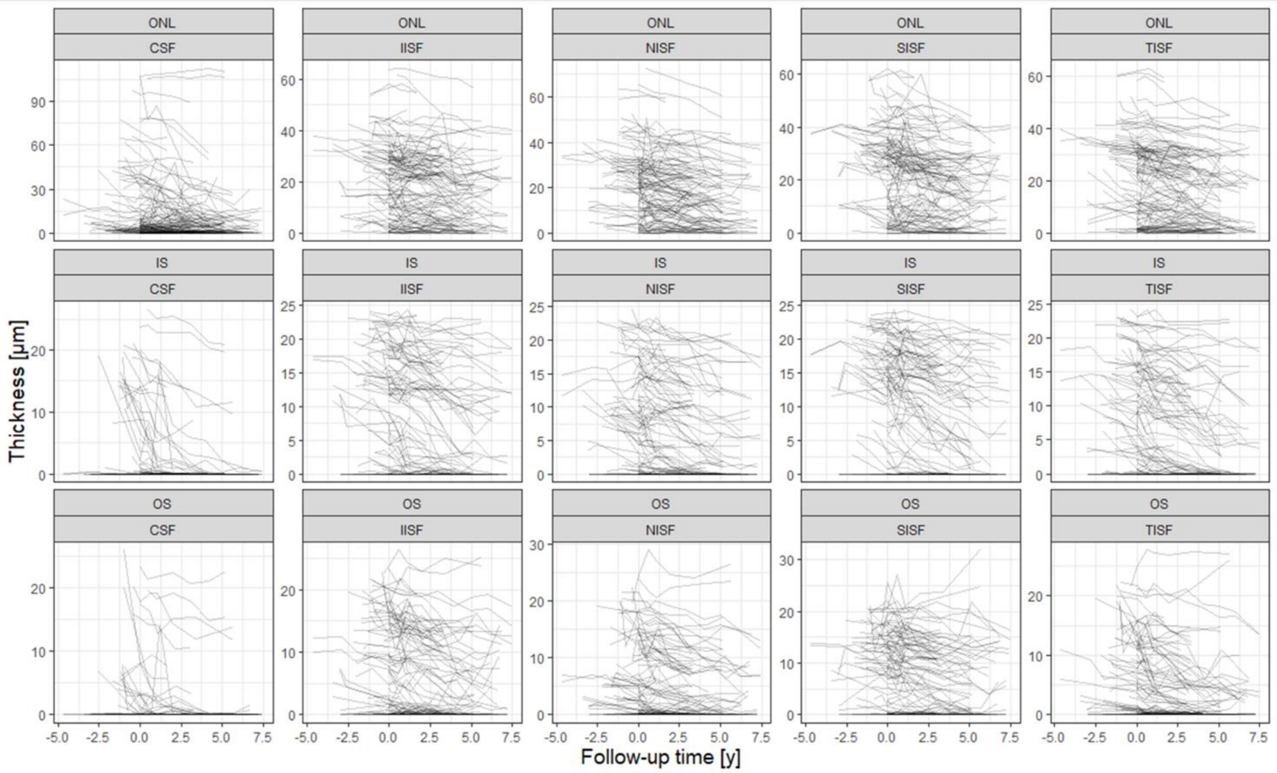

Abbreviations: Outer nuclear layer (ONL), inner segments (IS), outer segments (OS), central subfield (CSF), inferior inner subfield (IISF), nasal inner subfield (NISF), superior inner subfield (SISF), temporal inner subfield (TISF)

Supplementary Figure S8. Progression of inner retinal, retinal pigment epithelium, and choroidal degeneration over time

The line plots show the change in layer thicknesses (μm, y-axis) over time (x-axis) as a function of the retinal location (ETDRS subfields, panels). Please note, the *ETDRS-grid* is shown in Supplementary Figure S5.

These plots include data acquired prior to the baseline visit of the natural history study up to the last visit of each patient (N of patients = 66).

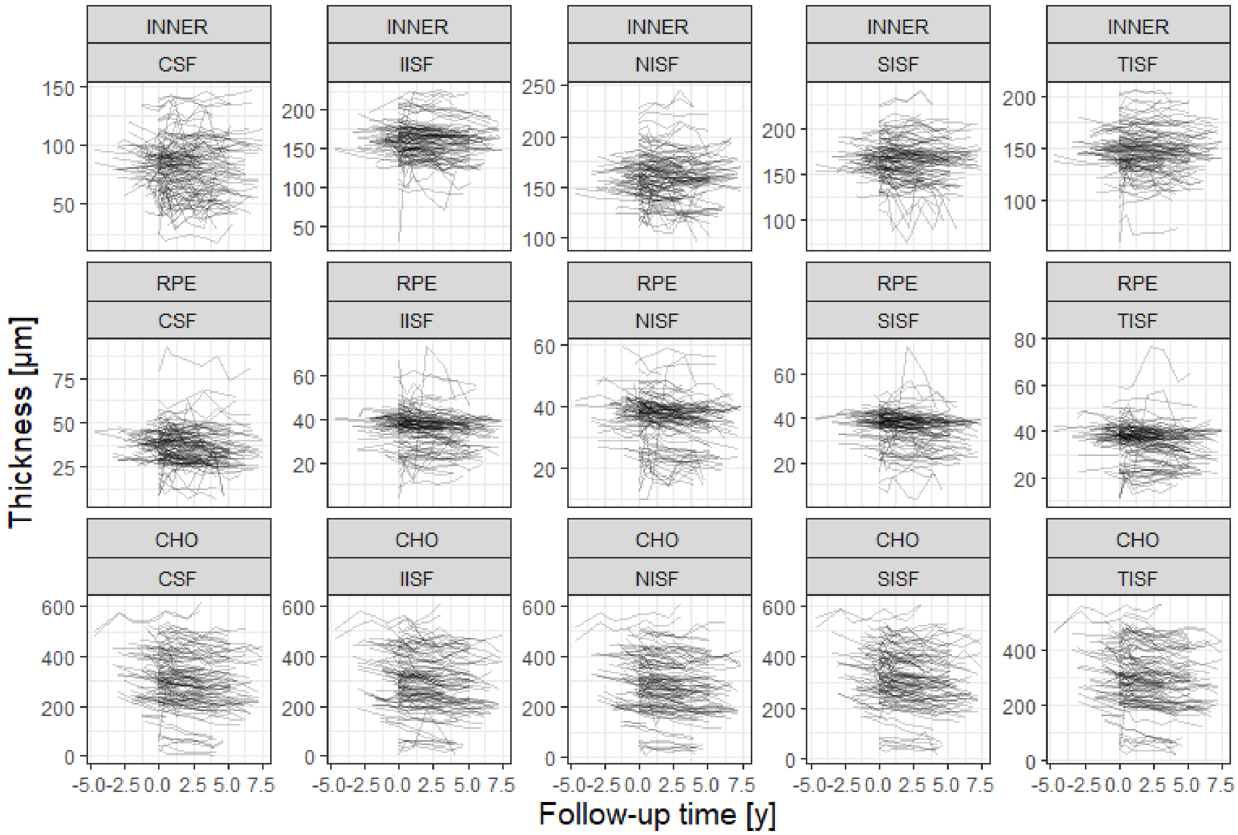

Abbreviations: Inner retina (INNER), retinal pigment epithelium (RPE), choroid (CHO), central subfield (CSF), inferior inner subfield (IISF), nasal inner subfield (NISF), superior inner subfield (SISF), temporal inner subfield (TISF)

Supplementary Figure S9. Estimation of the age of criterion ellipsoid zone (EZ) loss

The figure shows how the age of criterion *EZ-loss* (6.25 mm^2, horizontal black line) was estimated through linear regression of the square-root transformed *EZ-loss* area as a function of time. The right eye data from five patients is shown (patient ID indicated by the colors). The dots denote the measured area of *EZ-loss*, the lines show the fitted linear models, and the squares with the cross mark the estimated age of criterion *EZ-loss*.

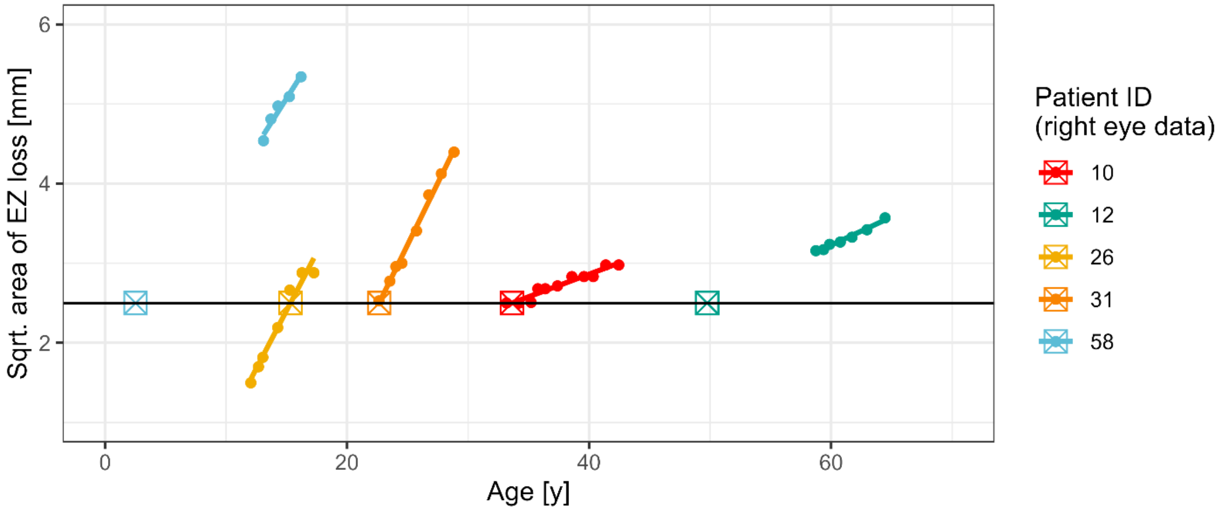

Supplementary Figure S10. Estimates for the age of criterion ellipsoid zone loss

(**A**) The first panel shows the age of criterion ellipsoid zone (EZ) loss from both eyes per patient (each patient represents a horizontal line). Noticeably, the age of criterion *EZ-loss* forms a cumulative Gaussian distribution function with no clustered groups.

(**B**) The second panel shows the estimates for the right eyes of patients (OD) plotted against the estimates for the left eyes (OS). Overall, the estimates from both eyes show a strong correlation with an (R^2) 90.7 %.

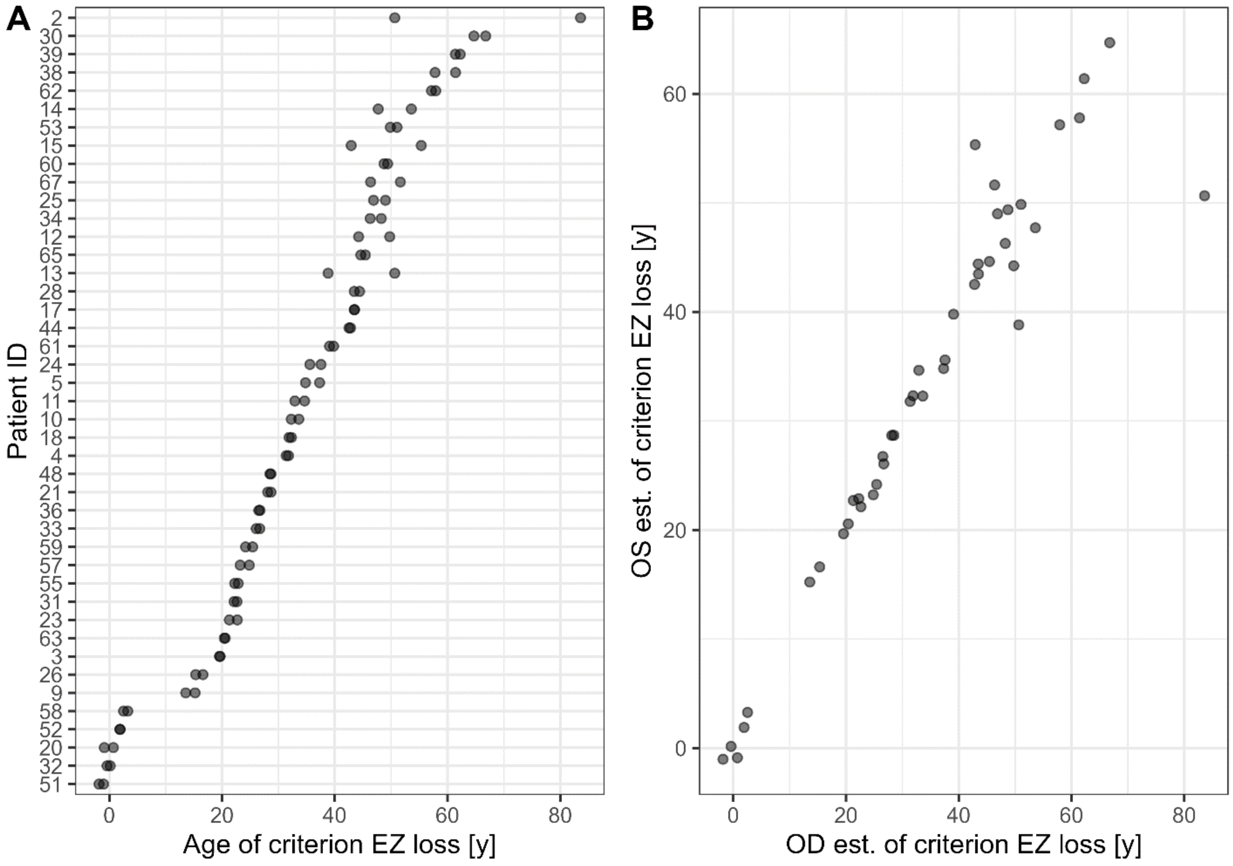

Supplementary Figure S11. Leave-one-out cross-validated (LOOCV) accuracy of the age of criterion ellipsoid zone loss

A subset of 23 patients was suitable to validate the additive model for predicting the age of criterion *EZ-loss* (AoC). These 23 patients had an overlap of both *ABCA4* variants with other patients. Accordingly, each training fold of 22 patients (n-1) provided estimates for the two variants of the one held-out patient. In this small sub-cohort, the additive model explained (LOOCV R^2) 24.1 % of the variability in AoC.

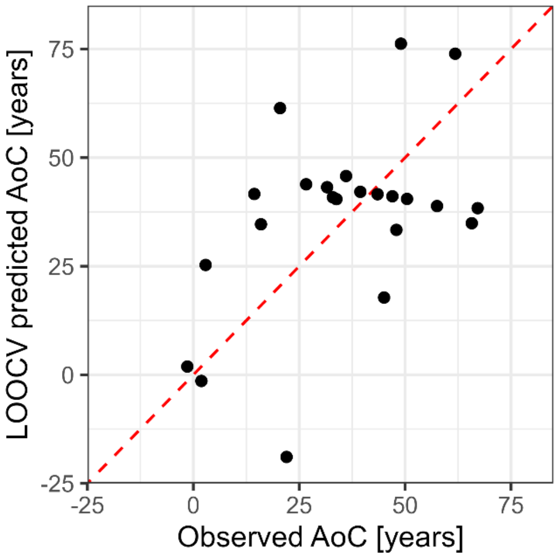

Supplementary Tables

Supplementary Table S1. Estimates for the Change in Retinal Layer Thickness Stratified by Contour-Line in z-score units

		0.43° contour line			1.29° contour line			2.58° contour line			5.16° contour line			7.73° contour line		
Layer	*Model Term*	*Estimate*	*SE*	*P*	*Estimate*	*SE*	*P*	*Estimate*	*SE*	*P*	*Estimate*	*SE*	*P*	*Estimate*	*SE*	*P*
INNER	(Intercept) [z-score units]	-1.538	0.139	<0.001	-1.222	0.114	<0.001	-0.877	0.1	<0.001	-0.389	0.096	<0.001	-0.108	0.087	ns
	Follow-up Time [z-score units per y]	0.028	0.017	ns	-0.022	0.017	ns	-0.033	0.016	<0.05	-0.013	0.012	ns	-0.023	0.007	<0.01
ONL	(Intercept) [z-score units]	-3.597	0.168	<0.001	-2.946	0.181	<0.001	-2.159	0.212	<0.001	-1.164	0.156	<0.001	-0.91	0.133	<0.001
	Follow-up Time [z-score units per y]	-0.14	0.021	<0.001	-0.1	0.018	<0.001	-0.064	0.016	<0.001	-0.033	0.015	<0.05	-0.01	0.013	ns
IS	(Intercept) [z-score units]	-3.337	0.424	<0.001	-2.57	0.385	<0.001	-1.439	0.373	<0.001	-0.48	0.089	<0.001	-0.233	0.076	<0.01
	Follow-up Time [z-score units per y]	-0.822	0.086	<0.001	-0.317	0.059	<0.001	-0.16	0.03	<0.001	-0.095	0.026	<0.001	-0.051	0.011	<0.001
OS	(Intercept) [z-score units]	-2.108	0.216	<0.001	-0.712	0.25	<0.01	-0.076	0.191	ns	0.263	0.084	<0.01	0.186	0.092	ns
	Follow-up Time [z-score units per y]	-0.591	0.051	<0.001	-0.371	0.062	<0.001	-0.189	0.056	<0.01	-0.067	0.037	ns	-0.015	0.017	ns
RPE	(Intercept) [z-score units]	0.615	0.164	<0.001	0.603	0.174	<0.001	1.184	0.182	<0.001	1.528	0.169	<0.001	1.515	0.157	<0.001
	Follow-up Time [z-score units per y]	0.034	0.049	ns	-0.003	0.037	ns	-0.111	0.027	<0.001	-0.086	0.019	<0.001	-0.08	0.012	<0.001
CHO	(Intercept) [z-score units]	-0.001	0.119	ns	0.009	0.12	ns	0.146	0.123	ns	0.309	0.113	<0.01	0.357	0.131	<0.01
	Follow-up Time [z-score units per y]	-0.058	0.009	<0.001	-0.06	0.009	<0.001	-0.054	0.008	<0.001	-0.052	0.01	<0.001	-0.042	0.009	<0.001

The estimates were obtained using linear mixed models (random intercept and slope models). The intercept may be interpreted as the average value at baseline. Degrees of freedom for P-values were computed using Satterthwaite's approximation.

Abbreviations: Outer nuclear layer (ONL), photoreceptor inner segments (IS), photoreceptor outer segments (OS), retinal pigment epithelium (RPE), choroid (CHO), not significant (ns)

Supplementary Table S2. Estimates for the Change in Retinal Layer Thickness Stratified by Contour-Line in µm

		0.43° contour line			1.29° contour line			2.58° contour line			5.16° contour line			7.73° contour line		
Layer	*Model Term*	*Estimate*	*SE*	*P*	*Estimate*	*SE*	*P*	*Estimate*	*SE*	*P*	*Estimate*	*SE*	*P*	*Estimate*	*SE*	*P*
INNER	(Intercept) [µm]	146.3	2.38	<0.001	153.79	2.86	<0.001	152.62	2.53	<0.001	159.76	5.13	<0.001	160.22	4.8	<0.001
	Follow-up Time [µm/y]	0.57	0.28	<0.05	-0.58	0.41	ns	-0.43	0.25	ns	-0.21	0.25	ns	-0.24	0.17	ns
ONL	(Intercept) [µm]	33.77	1.32	<0.001	35.24	1.55	<0.001	40.31	1.36	<0.001	42.72	1.03	<0.001	42.64	0.88	<0.001
	Follow-up Time [µm/y]	-1.22	0.18	<0.001	-0.87	0.13	<0.001	-0.55	0.13	<0.001	-0.31	0.12	<0.05	-0.1	0.1	ns
IS	(Intercept) [µm]	17.19	0.91	<0.001	17.79	0.95	<0.001	20.9	0.66	<0.001	22.31	0.19	<0.001	22.85	0.13	<0.001
	Follow-up Time [µm/y]	-1.41	0.15	<0.001	-0.59	0.1	<0.001	-0.3	0.07	<0.001	-0.19	0.05	<0.001	-0.1	0.02	<0.001
OS	(Intercept) [µm]	13.58	0.68	<0.001	17.19	0.83	<0.001	19.42	0.52	<0.001	20.04	0.23	<0.001	19.83	0.25	<0.001
	Follow-up Time [µm/y]	-1.67	0.14	<0.001	-1.05	0.17	<0.001	-0.53	0.15	<0.01	-0.15	0.09	ns	-0.04	0.05	ns
RPE	(Intercept) [µm]	38.04	0.58	<0.001	37.12	0.68	<0.001	39.23	0.6	<0.001	39.47	0.57	<0.001	39.29	0.5	<0.001
	Follow-up Time [µm/y]	0.1	0.16	ns	-0.05	0.11	ns	-0.38	0.08	<0.001	-0.32	0.06	<0.001	-0.28	0.05	<0.001
CHO	(Intercept) [µm]	286.65	13.35	<0.001	273.35	13.86	<0.001	281.86	12.53	<0.001	261.86	10.88	<0.001	252.12	9.78	<0.001
	Follow-up Time [µm/y]	-5.14	0.78	<0.001	-4.91	0.75	<0.001	-4.58	0.75	<0.001	-4.01	0.77	<0.001	-3.15	0.66	<0.001

The estimates were obtained using linear mixed models (random intercept and slope models). The intercept may be interpreted as the average value at baseline. Degrees of freedom for P-values were computed using Satterthwaite's approximation.

Abbreviations: Outer nuclear layer (ONL), photoreceptor inner segments (IS), photoreceptor outer segments (OS), retinal pigment epithelium (RPE), choroid (CHO), not significant (ns)

Supplementary Table S3. Changes in Retinal Layer Thickness Stratified by ETDRS Subfield

		Central subfield			Inferior inner subfield			Nasal inner subfield			Superior inner subfield			Temporal inner subfield		
Layer	*Model Term*	*Estimate*	*SE*	*P*	*Estimate*	*SE*	*P*	*Estimate*	*SE*	*P*	*Estimate*	*SE*	*P*	*Estimate*	*SE*	*P*
INNER	(Intercept) [μm]	83.12	2.7	<0.001	159.63	2.66	<0.001	160.22	2.69	<0.001	165.51	2.53	<0.001	147.51	2.49	<0.001
	Follow-up Time [μm/y]	-0.09	0.23	ns	-0.76	0.3	<0.05	-0.48	0.29	ns	-0.66	0.22	<0.01	-0.59	0.21	<0.01
ONL	(Intercept) [μm]	16.03	2.98	<0.001	20.84	1.78	<0.001	21.58	1.82	<0.001	23.87	1.85	<0.001	21.54	1.87	<0.001
	Follow-up Time [μm/y]	-1.22	0.32	<0.001	-0.69	0.21	<0.01	-0.82	0.17	<0.001	-0.8	0.18	<0.001	-0.74	0.18	<0.001
IS	(Intercept) [μm]	2.34	0.7	<0.01	8.32	1.04	<0.001	6.89	0.93	<0.001	10.02	1.05	<0.001	7.27	0.96	<0.001
	Follow-up Time [μm/y]	-0.4	0.12	<0.01	-0.47	0.07	<0.001	-0.47	0.06	<0.001	-0.46	0.07	<0.001	-0.46	0.07	<0.001
OS	(Intercept) [μm]	1.5	0.54	<0.01	6.4	0.89	<0.001	5.09	0.81	<0.001	7.78	0.88	<0.001	4.78	0.75	<0.001
	Follow-up Time [μm/y]	-0.15	0.05	<0.01	-0.37	0.09	<0.001	-0.37	0.09	<0.001	-0.3	0.08	<0.001	-0.41	0.1	<0.001
RPE	(Intercept) [μm]	37.45	1.17	<0.001	36.35	1	<0.001	36.5	0.94	<0.001	36.91	0.84	<0.001	36.3	0.96	<0.001
	Follow-up Time [μm/y]	-0.51	0.17	<0.01	-0.25	0.17	ns	-0.28	0.13	<0.05	-0.34	0.11	<0.01	-0.23	0.15	ns
CHO	(Intercept) [μm]	309.83	13.5	<0.001	303.52	13.87	<0.001	292.23	13.38	<0.001	320.19	12.91	<0.001	302.03	12.87	<0.001
	Follow-up Time [μm/y]	-6.71	0.91	<0.001	-6.81	0.84	<0.001	-6.37	0.88	<0.001	-7.05	0.93	<0.001	-6.74	0.81	<0.001

The estimates were obtained using linear mixed models (random intercept and slope models). The intercept may be interpreted as the average value at baseline. Degrees of freedom for P-values were computed using Satterthwaite's approximation.

Abbreviations: Outer nuclear layer (ONL), photoreceptor inner segments (IS), photoreceptor outer segments (OS), retinal pigment epithelium (RPE), choroid (CHO), not significant (ns)

Supplementary Table S4. Estimated age of criterion ellipsoid zone loss (AoC) for patients with two *ABCA4* variants

Patient ID	AoC (right eye) in years	AoC (left eye)	Variants 1	Change 1	Variants 2	Change 2
2	83.60121	50.64957	c.5882G>A	p.Gly1961Glu	c.4200C>A	p.Tyr1400*
3	19.55446	19.65455	c.2099G>A	p.Trp700*	c.4561C>T	p.Pro1486Leu
4	31.38156	31.79196	c.5882G>A	p.Gly1961Glu	c.666_678del13	p.Lys223Metfs14*
5	37.31315	34.80611	c.5461-10T>C		c.5603A>T	p.Asn1868Ile
9	13.5618	15.22601	c.6221dupG	p.Asn2075Glnfs*22	c.6079C>T	p.Leu2027Phe
10	33.62046	32.28024	c.5461-10T>C		c.2588G>C	p.Gly863Ala
11	32.91873	34.64664	c.5461-10T>C		c.2588G>C	p.Gly863Ala
12	49.75159	44.22958	c.5882G>A	p.Gly1961Glu	c.3210_3211dupGT	p.Ser1071Cys*14
13	50.63913	38.8138	c.5882G>A	p.Gly1961Glu	c.6229C>T	p.Arg2077Trp
14	53.58778	47.71052	c.5603A>T	p.Asn1868Ile	c.6112C>T	p.Arg2038Trp
15	42.91576	55.33742	c.5882G>A	p.Gly1961Glu	c.3364G>A	p.Glu1122Lys
17	43.49651	43.47128	c.5882G>A	p.Gly1961Glu	c.3050+5G>A	
18	31.89769	32.31428	c.768G>T	p.Val256Val	c.2966T>C	p.Val989Ala
20	0.723605	-0.88368	c.3113C>T	p.Ala1038Val	c.3113C>T	p.Ala1038Val
21	28.11156	28.67563	c.5222_5232del	p.Leu1741fs*	c.6729+61G>A	
23	21.29495	22.70735	c.5714+5G>A		c.161G>A	p.Cys54Tyr
24	37.56074	35.59066	c.3364G>A	p.Glu1122Lys	c.3385C>T	p.Arg1129Cys
25	46.87781	48.9966	c.5461-10T>C		c.2588G>C	p.Gly863Ala
26	15.32375	16.61564	c.5461-10T>C		c.634C>T	p.Arg212Cys
28	43.4599	44.39728	c.5461-10T>C		c.1762G>C	c.Asp576His
30	66.77655	64.69082	c.5461-10T>C		c.5603A>T	p.Asn1868Ile
31	22.64703	22.13294	c.5196+1G>A	Splice	c.6089G>A	p.Arg2030Gln
32	-0.39714	0.155911	c.2564G>A	p.Trp855*	c.868C>T	p.Arg290Trp
33	26.69393	26.06753	c.2588G>C	p.Gly863Ala	c.4139C>T	p.Pro1380Leu
34	48.24888	46.28208	c.2966T>C	p.Val989Ala	c.2385C>G	p.Ser795Arg
36	26.52343	26.73978	c.5882G>A	p.Gly1961Glu	c.5714+5G>A	
38	61.42521	57.78902	c.5603A>T	p.Asn1868Ile	c.214G>A	p.Gly72Arg
39	62.25483	61.38604	c.5882G>A	p.Gly1961Glu	c.5882G>A	p.Gly1961Glu
44	42.79987	42.52046	c.6089G>A	p.Arg2030Gln	c.4577C>T	p.Thr1526Met
48	28.49507	28.68105	c.5714+5G>A		c.4978C>T	p.Pro1660Ser
51	-1.8226	-1.01956	c.5461-10T>C		c.3259G>A	p.Glu1087Lys
52	1.913082	1.89073	c.5461-10T>C		c.3259G>A	p.Glu1087Lys
53	51.04451	49.85566	c.5461-10T>C		c.5603A>T	p.Asn1868Ile
55	22.25158	22.87328	c.5714+5G>A		c.5898+2T>C	
57	24.82991	23.22384	c.2588G>C	p.Gly863Ala	c.6449G>A	p.Cys2150Tyr
58	2.530374	3.274523	c.2564G>A	p.Trp855Ter	c.5461-10T>C	
59	25.43022	24.17841	c.5882G>A	p.Gly1961Glu	c.4661A>G	p.Glu1554Gly
60	48.73283	49.37154	c.5882G>A	p.Gly1961Glu	c.1937+1G>A	
61	39.06914	39.78622	c.5882G>A	p.Gly1961Glu	c.6088C>G	p.Arg2030Ter
62	57.90976	57.15737	c.5882G>A	p.Gly1961Glu	c.634C>T	p.Arg212Cys
63	20.39406	20.56414	c.5603A>T	p.Asn1868Ile	c.161G>A	p.Cys54Tyr
65	45.4351	44.62439	c.3322C>T	p.Arg1108Cys	c.6079C>T	p.Leu2027Phe
67	46.35726	51.63788	c.5603A>T	p.Asn1868Ile	c.3322C>T	p.Arg1108Cys

Appendix to the Methods

Section 1: Genotype-phenotype analysis

For the genotype-phenotype analysis, we included only patients with two pathogenic variants. Given the analysis approach (cf. below), patients were required to either (i) have at least one variant in common with other patients or (ii) bi-allelic identical mutations. Supplementary Figure S1 provides a detailed flow diagram regarding the inclusion/exclusion of patients.

For each eye, we computed the expected age of criterion *EZ-loss* (6.25 mm^2) using linear regression (Supplementary Figure S9). To achieve overlap among patients for the subsequent analysis, truncating and frameshift mutations were grouped as ‘null’ mutations, assuming that these do not result in a functional protein product (17). Based on previous work by Cideciyan and coworkers (17), we assumed that variants have an independent, additive contribution to the age of criterion *EZ-loss*. Accordingly, the following random intercept model was fit to the data for the genotype-phenotype analysis:

$$AoC_{ij} = 0 + \beta_1 x_{1i} + \beta_2 x_{2i} + \ldots + u_i + \varepsilon_{ij}$$

where

- $\boldsymbol{AoC_{ij}}$ represents the age of criterion *EZ-loss* for the *i*-th subject and *j*-th eye (i.e., OD or OS)
- $\boldsymbol{\beta_1}$ **to** $\boldsymbol{\beta_{28}}$ represent the regression coefficients for the *ABCA4* variants $x_1$to x_{28} (value range for each variant of 0 [absent], 1 [monoallelic], 2 [bi-allelic])
- $\boldsymbol{u_i}$ represents the random intercept for the *i*-th subject
- $\boldsymbol{\epsilon_i}$ represents the random residual for the *j*-th eye (i.e., OD or OS) in the *i*-th subject

Accordingly, the expected age of criterion *EZ-loss* for a given patient is the sum of the regression coefficients. A mixed model was applied given the repeated measures data (i.e., two estimates for the age of criterion *EZ-loss* for each patient [right and left eye]).

Heuristically, the model fitting can be (approximately) thought of as a stepwise process. The typical age of criterion EZ-loss for a patient with bi-allelic 'null' variants was 13.76 yr in our cohort. Accordingly, the coefficient for a single truncating mutation is 6.88 yr. Next, the severity of further variants in trans with a 'null' variant can be calculated. For example, the expected age of criterion EZ-loss of 41.51 yr for patients with p.Gly1961Glu in trans to a 'null' mutation can be used to derive the coefficient for p.Gly1961Glu of 34.63 yr (i.e., 41.51 yr - 6.88 yr). Thus, it is possible to derive a coefficient for all variants occurring either homozygously, or in trans with a variant present in other patients.

Section 2: Internal- and external validation of genotype-phenotype analysis results

We examined the error between predicted and observed age of criterion *EZ-loss* in our cohort using patient-wise leave-one-out cross-validation (LOOCV). To do so, c.5461-10T>C and c.5714+5G>A were added to the 'null' group given prior in vitro data (32), as well as the coefficients for the genotype-phenotype analysis observed herein. Thus, a subset of 23 patients was suitable for LOOCV due to the overlap of both *ABCA4* variants with other patients. Specifically, this means that each training fold of 22 patients (n-1) would provide estimates for the two variants of the one held-out patient.

The observed estimates of variant severity we calculated were compared to previous data from Cideciyan et al. 2009 (interval-scaled estimates for each variant for the delay of perimetry-based [retina-wide] sensitivity loss) (17), and data from Fakin et al. 2016 (ordinal-scaled categorization based on ERG characteristics) (4).
